# Low-dose nivolumab with neoadjuvant chemotherapy and oral metronomic therapy in borderline resectable oral cavity squamous cell carcinoma: a phase II trial

**DOI:** 10.1016/j.lansea.2026.100743

**Published:** 2026-03-05

**Authors:** Praveen Kumar Marimuthu, Chitra Renukaradhya, Shalini Sahu, Ajoy Oommen John, Amit Jiwan Tirkey, Balu Krishna Sasidharan, Konduru Vidya, Jeyashanth Riju, Mansi Agrawal, Meera Thomas, Jino Victor Wilson, Anjana Joel, Sharief K. Sidhique, Josh Thomas Georgy, Divya Bala Thumaty, Deepa Susan Joy Philip, Kovilapu Harikrishna, Anil Kumar Subbarao, Manu Mathew, Simon Pradeep Pavamani, Rajiv C. Michael, Rajesh Isiah, Jansi Rani, Ashish Singh

**Affiliations:** a Department of Medical Oncology, Christian Medical College Vellore, India; b Department of General Pathology, Christian Medical College Vellore, India; c Department of Radiology, Christian Medical College Vellore, India; d Department of Head and Neck Surgery, Christian Medical College Vellore, India; e Department of Radiation Oncology, Christian Medical College Vellore, India; f Department of Biostatistics, Christian Medical College Vellore, India

**Keywords:** Oral cavity squamous cell carcinoma (OSCC), Borderline resectable, Neoadjuvant therapy, Nivolumab, Oral metronomic therapy, Pathological response

## Abstract

**Background:**

Non-surgical management of oral cavity squamous cell carcinoma (OSCC) has poorer outcomes compared to surgery. In borderline resectable tumors, historical neoadjuvant chemotherapy achieves surgical conversion in only about 40%. Combining low-dose immunotherapy and oral metronomic therapy (OMT) with chemotherapy may enhance resection rate and survival.

**Methods:**

Between April 2023 and April 2024, patients deemed ‘borderline resectable’ OSCC based on predefined criteria by a multidisciplinary tumor board were prospectively offered this Phase II single-arm interventional trial setting. Patients received two 21-day cycles of carboplatin, nab-paclitaxel, low-dose nivolumab, and six weeks of erlotinib, methotrexate, celecoxib, with additional cycle(s) if needed. Primary endpoint was R0 resection rate. Secondary endpoints were objective response rate, pathologic response, safety, event-free survival (EFS), and overall survival (OS). Immune biomarkers and Volumetric assessment were exploratory endpoints. The trial was prospectively registered in the Clinical Trial Registry of India (CTRI/2023/04/051617).

**Findings:**

Of 34 patients, all except one completed planned neoadjuvant therapy. After 2 cycles, 22 (66·6%) had partial response and 11 had stable disease; none progressed. Twenty six underwent surgery; 25 achieved R0 resection (25/33–75·7% conversion). Four of seven remaining patients received additional cycle(s); three subsequently achieved R0 resection. Overall conversion rate was 90·3% (28/31) excluding 2 patients who refused further treatment. Major pathological response occurred in 12 patients (41·4%), including four with pathological complete response. Grade ≥3 toxicities occurred in 5 of 34 patients (14·7%), with no treatment-related deaths. One patient had grade 4 diarrhea with grade 4 acute kidney injury.

**Interpretation:**

The NeoLOCUS regimen offers an affordable, outpatient chemo-immunotherapy approach that improves surgical conversion and pathological response in borderline resectable OSCC.

**Funding:**

Fluid Research Grant- 10.13039/501100005918Christian Medical College, Vellore, India.

**Research in context**

**Evidence before this study**

Borderline resectable oral cavity squamous cell carcinoma (OSCC) remains a major therapeutic challenge in low- and middle-income countries, where many patients present with locally advanced disease and curative options are limited. We searched PubMed, Embase, the Cochrane Library, ClinicalTrials.gov, and regional oncology meeting abstracts from Jan 1, 1985, to May 31, 2025, without language restrictions, using combinations of the terms “oral cavity squamous cell carcinoma,” “borderline resectable,” “neoadjuvant chemotherapy,” “nivolumab,” and “oral metronomic therapy.” Eligible studies reported outcomes of neoadjuvant systemic therapy in borderline or technically unresectable OSCC. Triplet chemotherapy regimens improve operability but are associated with high toxicity. Platinum-based doublets combined with oral metronomic therapy achieve resectability with a lower toxicity burden and are feasible in resource-constrained settings. However, whether the addition of immune checkpoint blockade could enhance these outcomes has not been prospectively tested. Evidence for PD-1 blockade in this context is limited to studies in resectable OSCC or in recurrent/metastatic head and neck cancers. We did not identify any prospective trials evaluating platinum doublets plus oral metronomic therapy with immunotherapy in borderline resectable OSCC. Most available studies were retrospective, single-center, and heterogeneous, precluding pooled analyses.

**Added value of this study**

This phase II trial is a prospective study to evaluate a short-course, fully outpatient neoadjuvant regimen combining low-dose nivolumab, carboplatin, nab-paclitaxel, and oral metronomic methotrexate, celecoxib, and erlotinib in borderline resectable OSCC. The regimen achieved a 75% rate of R0 resection after just two cycles and 90% overall, with major pathological response rates surpassing historical benchmarks with chemotherapy alone. By incorporating tumor immune microenvironment profiling and volumetric imaging, the study provides novel biological and radiological correlates of response, with particular relevance for high-burden, resource-constrained settings.

**Implications of all the available evidence**

Our findings suggest that an affordable, outpatient chemo-immunotherapy regimen can substantially improve curative surgery rates with acceptable toxicity in borderline resectable OSCC. This approach directly addresses treatment gaps in low- and middle-income countries, where oral cavity cancers are prevalent and resources for prolonged inpatient therapy are scarce. Taken together with prior evidence, these results support prioritization of larger multicentre randomized trials to validate long-term survival outcomes, refine patient selection, and inform regional policies on integrating cost-effective immunotherapy into standard care.

## Introduction

Oral cancers are among the most common malignancies in South-Central Asia, Southeast Asia, and Melanesia, contributing significantly to the cancer burden in these regions. This high prevalence is largely attributed to the widespread use of smokeless tobacco and areca nut, especially in lower income- and middle-income countries (LMICs).^1^, ^2^, ^3^, ^4^ Most OSCCs arise in the buccal mucosa or anterior tongue, with surgical resection (R0) being the mainstay of treatment. However, most present at Stage IV with a 55% 5-year OS,^5^ and only 25% are amenable to R0 resection.^6^ Incomplete resection with positive/close margins reduces disease-free survival by 20% and overall survival by 13%.^7^^,^^8^ Absolute contraindications for surgery are extension of tumor to the base of skull, prevertebral fascia or encasement of the carotid.^9^ However, there exists a proportion of patients with involvement of other anatomical landmarks which confer a higher likelihood of positive resection margins or debilitating functional loss. The term “Borderline resectable” OSCC has been used to describe this group. Patil and colleagues^10^ proposed a criterion for this entity, and subsequent studies have attempted to refine these definitions to identify favorable patient subgroups within these categories.^11^

In borderline resectable OSCC, non-surgical modalities such as external beam radiotherapy (EBRT), with or without concurrent chemotherapy, are often used as definitive or sequential therapy after neoadjuvant chemotherapy (NACT). Multiple studies have shown that non-surgical management yields inferior outcomes compared to surgery.^10^^,^^12^, ^13^, ^14^ Achieving surgical resectability through effective neoadjuvant therapy is therefore a critical goal in borderline resectable OSCC.

Three-drug NACT regimens like TPF (docetaxel, cisplatin, and 5-fluorouracil) achieve higher conversion to resectability and improved survival compared to doublets PF (cisplatin and 5-fluorouracil) or TP (cisplatin and paclitaxel).^15^, ^16^, ^17^, ^18^, ^19^, ^20^, ^21^ However, the significant toxicity and resource demands of TPF limit its applicability in routine practice, and only about one-third of eligible patients receive it, often with dose reductions.^21^

Immune checkpoint inhibitors (ICIs) improve outcomes in recurrent or metastatic (R/M) HNSCC,^22^, ^23^, ^24^, ^25^ but remain prohibitively expensive in resource-limited settings.^26^ A randomized trial by Patil and colleagues demonstrated that Low-dose nivolumab (LD-Nivo) plus oral metronomic therapy (OMT) improved survival in R/M HNSCC.^27^ At our center, combining LD-Nivo with 2- or 3-drug NACT rendered about one-third of patients with borderline-resectable OSCC eligible for surgery.^28^ Emerging evidence suggests that adding metronomic components to NACT doublets may improve response and resectability.^29^^,^^30^

There remains an urgent need for a neoadjuvant regimen that is effective, affordable, and well tolerated, while maximizing the likelihood of curative resection in borderline resectable OSCC. This study was undertaken to evaluate the safety and efficacy of a short-course, multidrug regimen combining NACT, OMT, and LD-Nivo in this challenging patient population. This approach has a strong biologic rationale - NACT provides rapid cytoreduction and induces immunogenic cell death, OMT exerts anti-angiogenic and immune-modulatory effects,^31^^,^^32^ and LD-Nivo enhances antitumor immunity through checkpoint blockade.^33^^,^^34^ Their distinct mechanisms of action and non-overlapping toxicity profiles enable safe combination while simultaneously targeting multiple tumor-promoting pathways.

## Methods

**Fig. 1:**
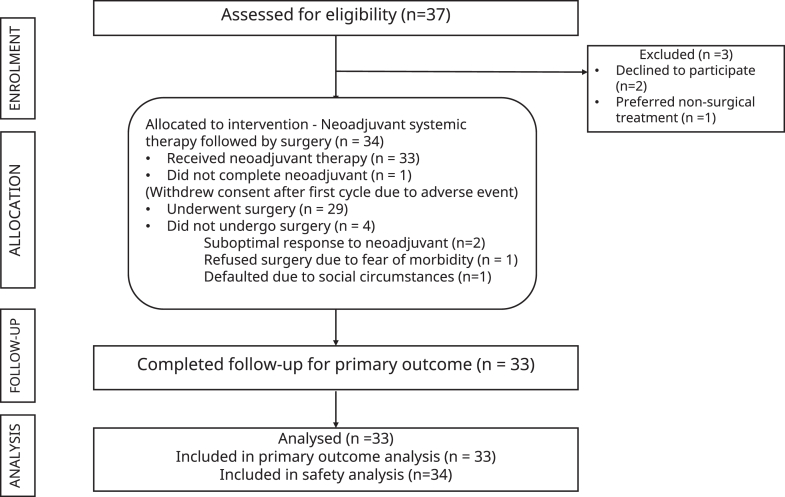
Study flow diagram of the NeoLOCUS trial.

We conducted a prospective, single-arm, phase II study at a tertiary referral center, recruiting eligible patients between April 2023 and April 2024. Data cut-off for analysis was 14th June 2025. Fig. 1 outlines the participant flow.

Adult patients who had biopsy-proven OSCC with an ECOG performance status of 0–1 and deemed ‘borderline resectable’ based on predefined criteria established by our Head and Neck Multidisciplinary Tumor Board (HN-MDT) were screened for enrollment. The criteria for borderline resectability included–a. Buccal mucosa primary with diffuse margins and peritumoral extension (edema) reaching up to the level of the sigmoid notch of the mandible but not above. b. Oral tongue primary with extension to the posterior one-third of the tongue, with peritumoral edema extending up to the hyoid bone. c. Oral cavity tumors of any subsite with extensive skin involvement confined to the surgical site.

Detailed inclusion and exclusion criteria are provided in the protocol document.

All patients received neoadjuvant chemoimmunotherapy consisting of: Intravenous Carboplatin AUC5 Day 1+ Intravenous nab-paclitaxel 200 mg/m^2^ Day 1 with maximum dose capped at 300 mg + Intravenous low-dose Nivolumab (Weight based dosing: <65 kg = 20 mg; ≥65 kg = 40 mg) Day 1 every 21 days for 2 cycles with OMT (Methotrexate 9 mg/m^2^ once weekly + Celecoxib 200 mg twice daily + Erlotinib 100 mg once daily) for 6 weeks. Treatment modifications (dose reductions/delays) and discontinuation criteria are detailed in the protocol document. All patients had scheduled toxicity assessments on Day 10 and Day 20 of each cycle, with instructions to report intercurrent symptoms between visits.

Two weeks after completing neoadjuvant therapy, patients underwent repeat clinical reassessment by the operating surgeon and cross-sectional imaging (MRI for tongue and CT for buccal mucosa primaries). Resectability decisions were made by the HN-MDT. Patients deemed resectable and taken for surgery were considered conversions. The surgical margins were taken based on the post-neoadjuvant residual tumor, as assessed clinically and radiologically. Patients deemed unresectable after 2 cycles were managed as per HN-MDT recommendations, which included additional cycles of neoadjuvant therapy or definitive chemoradiation based on response.

The primary outcome was the surgical conversion rate, defined as the proportion of patients achieving R0 resection following neoadjuvant therapy.

### Secondary outcomes

#### Safety

Incidence of clinical and laboratory adverse events, graded per CTCAE v5·0. All patients who received at least one cycle of neoadjuvant therapy were included in safety analyses.

#### Objective response rate (ORR)

Radiological responses were assessed after 2 cycles according to RECIST v1·1 (based on the sum of diameters (SODs) of the primary tumor (longest diameter) and/or short axis diameter of lymph nodes) by a radiologist and categorized as complete response (CR), partial response (PR), stable disease (SD), or progressive disease (PD).

#### Pathological response rate

Pathological responses were evaluated in resected specimens of both primary tumors and lymph nodes. Responses were classified as pathological complete response (pCR; no residual viable tumor), major pathological response (MPR; ≤10% viable tumor cells), and non-MPR (>10% viable tumor cells). For exploratory analyses, patients were stratified into good responders (MPR: pCR, MPR: Non-pCR) and poor responders (non-MPR).

#### Survival endpoints

##### Event-free survival (EFS)

Time from diagnosis to progression, recurrence, second primary malignancy, or death from any cause. Patients without events were censored at last follow-up.

##### Overall survival (OS)

Time from diagnosis to death from any cause. Patients alive at analysis were censored at last follow-up.

#### Exploratory outcomes

##### Tumor immune microenvironment (TiME) profiling

Pre-treatment profiling was performed on biopsy specimens. Stromal tumor-infiltrating lymphocytes (sTILs) were assessed using International Immuno-Oncology Biomarker Working Group guidelines.^35^ sTILs were scored as mean percentages across five high-power fields (HPFs) in 5–10% increments. Immunohistochemistry (IHC) quantified CD8^+^ (cytotoxic), FOXP3^+^ (regulatory), and CD4^+^ (helper) T-cell subsets, with immune cell counts reported as absolute numbers per HPF (Supplemental Figure S1A). Two pathologists independently assessed all slides; discrepancies were resolved by consensus.

Post-treatment profiling was performed on resection specimens using the same markers and methodology (Supplemental Figure S1B). Patients with pCR were excluded from CD8/FOXP3 ratio analysis (no residual tumor). For FOXP3^+^ Treg comparisons, patients with pCR were included with post-treatment Treg counts set to zero. TiME profiling was introduced as a protocol amendment.

##### Volumetric response evaluation criteria in solid Tumors (vRECIST)

Contrast-enhanced CT scans (2·5 mm slice thickness) and, for oral tongue primaries, fused MRI sequences were imported into Eclipse v16·1 (Siemens Healthineers, Germany). A head-and-neck–trained radiation oncologist manually segmented the gross tumor volume (GTV) on each scan, delineating the primary lesion and the two largest cervical lymph nodes judged significant by multidisciplinary team (MDT) consensus based on size, morphology, and radiologic features. MDT consensus was used instead of the strict RECIST short-axis threshold (>1·5 cm) to reflect real-world gross nodal contouring. At each time point, individual GTVs were summed to derive total tumor volume (TTV). Volumetric response was defined based on the percentage change in TTV from baseline to after two cycles of neoadjuvant therapy. vRECIST response categories were: Volumetric partial response (vPR):≥65% decrease in TTV; Volumetric stable disease (vSD):change between −65% and <+40%; Volumetric progressive disease (vPD): ≥40% increase in TTV.

### Statistical analysis

The primary endpoint was evaluated using Fleming's optimal two-stage design,^36^ testing the null hypothesis of a true surgical conversion rate of 40% against a one-sided alternative. In stage one, 11 patients were accrued. Predefined stopping rules specified study termination for futility if ≤ 5 conversions occurred, or early rejection of the null hypothesis if ≥ 10 conversions were achieved. Otherwise, 20 additional patients were enrolled to reach a total of 31. The null hypothesis was rejected if ≥ 17 conversions were observed, providing a type I error of 0·0491 and 81% power at a true conversion rate of 65%. A 10% attrition rate was incorporated, adjusting the sample size to 34 patients. Interim analysis results were reviewed by the Data Safety Monitoring Board (DSMB) after stage one accrual.

All analyses were conducted using R (version 2025·05·1 + 513).Descriptive statistics were used to summarize patient demographics, clinical characteristics, and treatment outcomes. Categorical variables were compared using Chi-square or Fisher's exact tests, and continuous variables were compared using Mann–Whitney U tests or t-tests, as appropriate. Paired comparisons of pre- and post-treatment immune markers were performed using the Wilcoxon signed-rank test. Survival outcomes, including event-free survival (EFS) and overall survival (OS), were estimated using the Kaplan–Meier method, with group comparisons performed using the log-rank test. Adverse events were summarized as counts and percentages by CTCAE v5·0 grade and system organ class. A two-sided p < 0·05 was considered statistically significant. Data visualization was performed primarily with *ggplot2*, *ComplexHeatmap*, and *survminer*.

### Ethics statement

Institutional Review Board (IRB) approval was obtained (IRB Min No. 15148 dated 25·01·2023), and the study was registered in the Clinical Trial Registry of India (CTRI/2023/04/051617). Written informed consent was obtained from all participants before enrollment.

### Role of the funding source

This was an investigator-initiated trial funded by an institutional Fluid Research Grant following approval by the Institutional Review Board. No external funding was received for the conduct of the trial or for the preparation of the manuscript.

## Results

At the interim analysis after the first 11 patients, 7 underwent R0 resection after 2 cycles. Having met the predefined criteria for continuation, the study proceeded to enroll the remaining cohort as per the two-stage design. Baseline characteristics of the 34 enrolled patients are summarized in Table 1. All but one patient completed the planned neoadjuvant therapy.

**Table 1:** Baseline characteristics.

Parameter	VALUE (N = 34)
Patient-Related	
Median (IQR) Age	44·5 (38–54)
Gender	
Male	31 (91·2%)
Female	3 (8·8%)
ECOG PS 1	34 (100%)
Tobacco use	
Chewing only	18 (53%)
Smoking only	5 (14·7%)
Chewing and smoking	3 (8·8%)
None	8 (23·5%)
Comorbidities	
Diabetes Mellitus	1 (2·9%)
Systemic Hypertension	6 (17·6%)
Nil	27 (79·5%)
Tumor-Related	
Subsite	
Oral Tongue	17 (50%)
Buccal Mucosa	17 (50%)
Histology grade	
Well-differentiated	7 (20·6%)
Moderately differentiated	23 (67·6%)
Poorly differentiated	4 (11·8%)
Stage	
III	1 (2·9%)
IVA	15 (44·1%)
IVB	18 (53%)
Tumor Micro-Environment Related	*Median (IQR) (n = 27)*
CD4+ T cells	10·0 cells/hpf (5·0–10·0)
CD8+ T cells	5·0 cells/hpf (5·0–10·0)
FOXP3+ Tregs	1·0 cells/hpf (1·0–1·0)
CD8/FOXP3 ratio	5·0 (3·5–5·0)
Treatment-Related	
Cumulative Nivolumab Dose	
Median (IQR)	80 (40–80)
40 mg	14
>40 mg	19

After two cycles, 22 patients achieved a partial response and 11 had stable disease, yielding an objective response rate (ORR) of 66·6% (RECIST v1·1). No patient experienced disease progression. Correlation analysis revealed that ORR was significantly associated with clinical stage (p < 0·001, Chi-square test): patients with Stage IVA disease more frequently achieved partial responses, while those with Stage IVB had predominantly stable disease. Similarly, tumor subsite correlated significantly with ORR (p < 0·001, Chi-square test): oral tongue tumors responded more frequently with partial responses, whereas buccal mucosa tumors showed more stable disease. Cumulative nivolumab dose group (40 mg vs > 40 mg) was not significantly associated with ORR (p = 0·383, Chi-square test).

**Fig. 2:**
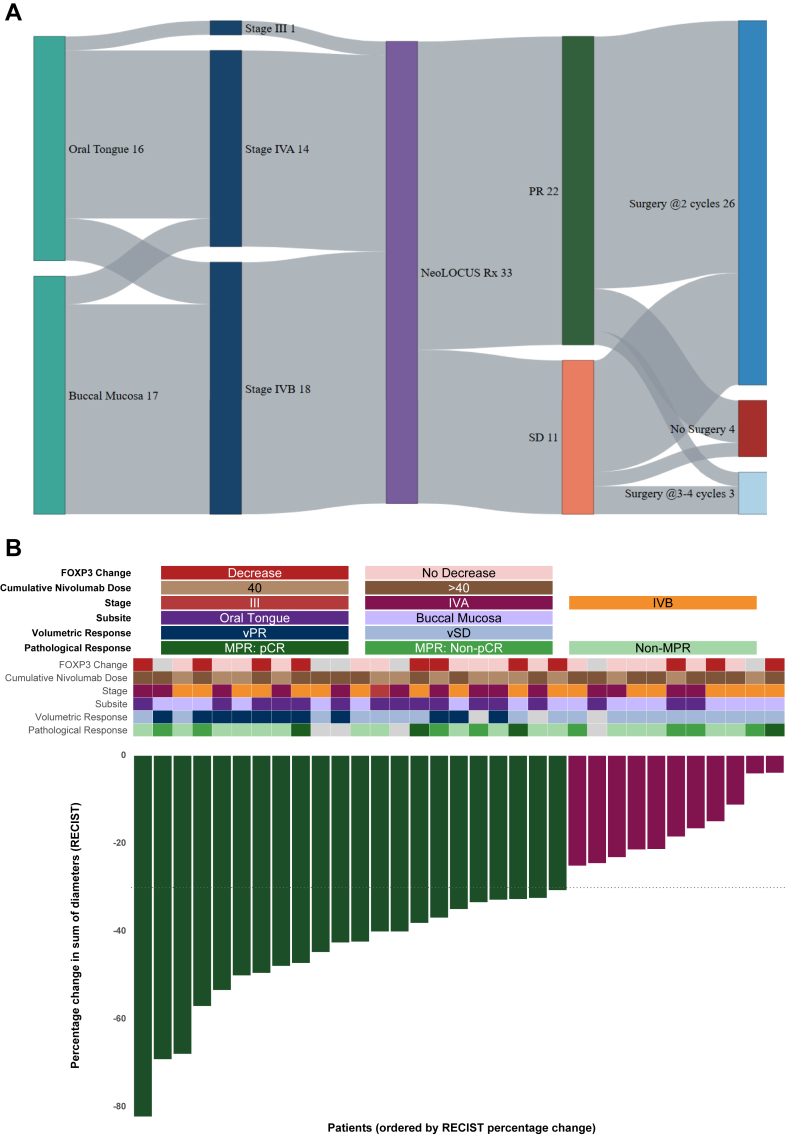

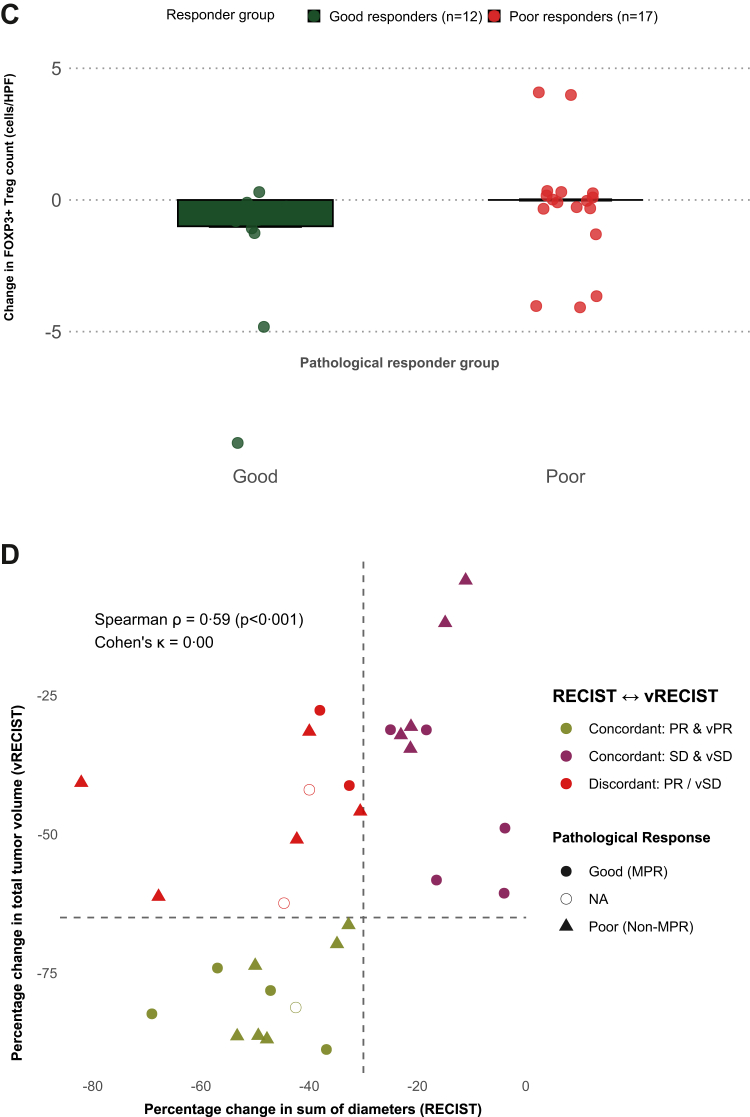
A. Patient flow through neoadjuvant treatment and subsequent management in the NeoLOCUS trial. B. Integrated clinical, radiological, pathological, and immunological landscape of patients treated in the NeoLOCUS trial. Waterfall plot showing percentage tumor size change per patient, with bar colors indicating radiologic response (olive green: partial response; maroon: stable disease). The stacked oncoprint heatmap depicts FOXP3 change, cumulative nivolumab dose, baseline stage, tumor subsite, volumetric response, and pathological response. Gray tiles denote missing data. C. Change in intratumoral FOXP3^+^ regulatory T-cell counts by pathological responder group. Good responders (N = 12) are shown in dark green and poor responders (N = 17) in red. Individual patient-level changes in FOXP3^+^ regulatory T-cell density (cells per high-power field) from baseline to post-neoadjuvant therapy are shown, stratified by pathological response category. D. Agreement between RECIST and volumetric response (vRECIST) in the NeoLOCUS trial (N = 30). Comparison of radiological response categories defined by RECIST 1.1 and volumetric response assessment (vRECIST), illustrating concordant and discordant classifications. Thresholds at −30% (RECIST PR) and −65% (vPR) shown as dashed lines. Colors indicate concordance/discordance; shapes denote pathological responder status.

Twenty-six patients proceeded to surgery after two cycles; of these, 25 underwent R0 resection, corresponding to a surgical conversion rate of 75·7% (25/33). One patient had an R1 resection (soft tissue margin). Among the seven patients with suboptimal response on clinical examination and imaging following two cycles, four received additional cycles of neoadjuvant therapy after re-evaluation by the Head and Neck Multidisciplinary Tumor Board (HN-MDT), and three subsequently underwent R0 resection. Of the remaining four patients, two received definitive chemoradiation due to suboptimal response to neoadjuvant therapy, one declined both surgery and radiotherapy, opting for oral metronomic therapy alone, and one defaulted due to social circumstances. This brought the overall surgical conversion rate to 90·3% (28/31). The sequence of events is illustrated in Fig. 2A.

Pathology was evaluable in 29 patients. MPR was observed in 12 (41·4%), including 4 (13·8%) with pCR; 17 (58·6%) had non-MPR. Supplementary Table S1 details pathological responses overall and at primary and nodal sites. Baseline factors including stage, subsite, and cumulative nivolumab dose were not significantly associated with pathological response (all p ≥ 0·716; odds ratios 0·70–1·19).

Immune profiling was performed in patients who underwent surgery to enable paired pre- and post-treatment comparisons. At baseline, 27 of 29 eligible patients had sufficient tissue for analysis; two were excluded due to inadequate archived material. Post-treatment profiling was available for 20 patients; seven patients with pCR at the primary site were excluded from post-treatment immune assessment due to lack of residual tumor (Supplementary Tables S2A and S2B).

Comparisons of immune biomarkers before and after therapy, assessed using the Wilcoxon signed-rank test, revealed no significant changes in the overall cohort. Median stromal TILs increased from 10·0% to 15·0% (p = 0·347); median CD4 from 10·0 to 12·5 cells/HPF (p = 0·061); and median CD8 from 5·0 to 7·5 cells/HPF (p = 0·330). FOXP3 median values remained 1·0 across both time points (baseline IQR 1·0–1·0, post-treatment IQR 0·5–1·0, p = 0·051). The CD8/FOXP3 ratio showed no significant change (median 5·0; p = 0·277).

When stratified by response, FOXP3 levels showed a significant reduction among good responders (median Δ −1·0; IQR –2·0 to 0·0; p = 0·014), but not among poor responders. The magnitude of FOXP3 reduction (ΔFOXP3) was significantly greater in good responders than in poor responders (median Δ −1·0 vs 0·0; IQR −2·0 to 0·0 vs −1·0 to 1·0; U = 46·0, p = 0·036, Mann–Whitney U test). No significant changes were observed for TILs, CD4, CD8, or CD8/FOXP3 ratio in either subgroup.

Fig. 2B integrates ORR with key clinical, pathological, and immune biomarker variables, and Fig. 2C illustrates changes in FOXP3^+^ Treg counts between baseline and post-therapy in good and poor responders.

Among 30 evaluable patients with paired baseline and post–two-cycle imaging, the median total tumor volume (TTV) decreased from 35·85 cm^3^ (IQR 28·33–52·73) at baseline to 17·10 cm^3^ (IQR 8·55–25·25) after neoadjuvant therapy, a median percentage change of −54·6% (IQR −73·99 to −32·74). Eleven patients (36·7%) achieved volumetric PR (vPR) and 19 (63·3%) had volumetric stable disease (vSD); no vPD events were observed. Volumetric percentage change correlated moderately with change in RECIST sum of diameters (Spearman ρ = 0·593; p = 0·0006). Agreement between RECIST and vRECIST classifications for PR vs SD was moderate (Cohen's κ = 0·449), with reclassification in 9 of 20 RECIST PRpatients all to vSD (Fig. 2D, Supplemental Figure S2).

Neither volumetric (vRECIST) nor linear (RECIST) radiological response significantly predicted pathological response. The odds of being a good responder were similar for vPR versus vSD (OR 0·96; 95% CI 0·23–4·02; p = 0·956) and for RECIST PR versus SD (OR 0·58; 95% CI 0·12–2·75; p = 0·694).

Using the reassessment scan date as the baseline landmark, vRECIST showed no association with event-free survival, with similar 12-month EFS for vPR and vSD (72·7% vs 73·0%; log-rank p = 0·956). RECIST categories likewise did not stratify EFS (12-month EFS 76·8% vs 63·6% for PR vs SD; log-rank p = 0·423).

**Fig. 3:**
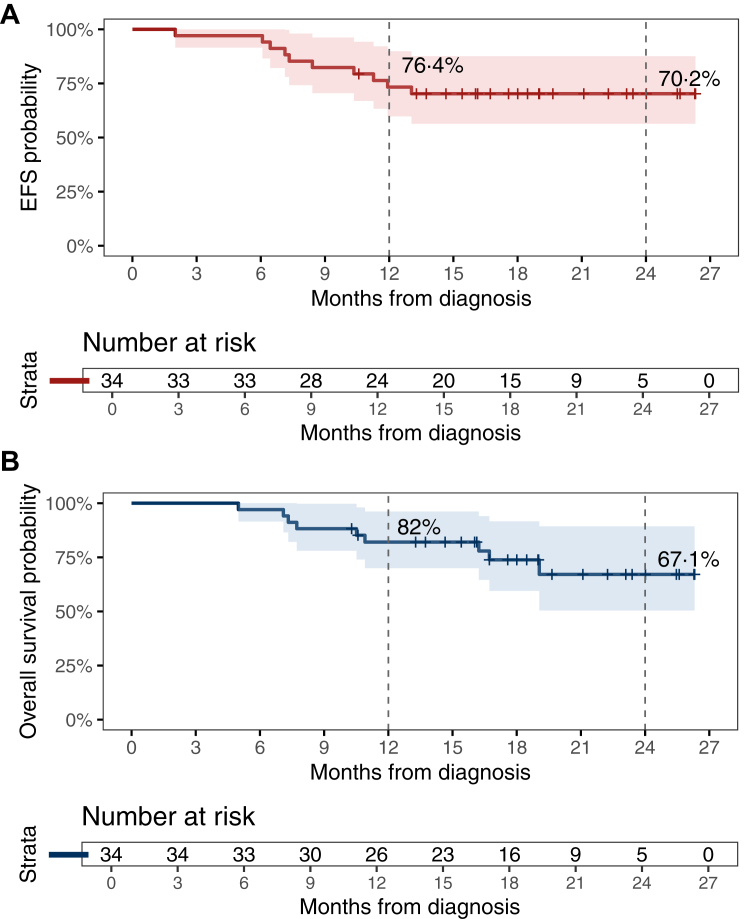
A. Event-free survival (EFS) in the neolocus cohort. Kaplan–Meier estimates of event-free survival from the time of diagnosis. Events were defined as disease progression, recurrence, or death from any cause. B. Overall survival (OS) in the neolocus cohort. Kaplan–Meier estimates of overall survival from the time of diagnosis. Death from any cause was considered an event. Note: All deaths were counted as events for both EFS and OS if an EFS event had not previously occurred. The numerically lower estimated overall survival compared with event-free survival at the 24-month landmark reflects heavy censoring and a limited number of patients at risk at later follow-up and should be interpreted with caution.

At a median follow-up of 18 months (IQR 14·7–21·1), median event-free survival (EFS) and overall survival (OS) were not reached. Ten of 34 patients (29·4%) experienced an EFS event, with estimated EFS rates of 76·4% (95% CI, 62·0–87·0%) at 1 year and 70·2% (95% CI, 49·5–84·8%) at 2 years (Fig. 3A). Among the ten events, seven were local or regional progression, one was distant progression, one was combined locoregional and distant progression, and one was death without documented progression. Nine patients (26·5%) had died by data cut-off, with OS rates of 82·0% (95% CI, 65·2–91·9%) at 1 year and 67·1% (95% CI, 45·5–82·9%) at 2 years (Fig. 3B).

No treatment-related deaths occurred. Grade ≥3 toxicities were reported in 5 of 34 patients (14·7%). One patient developed Grade 4 diarrhea with concurrent Grade 4 acute kidney injury after the first cycle, withdrew consent upon recovery, and defaulted further treatment. Grade 3 events included anemia (n = 3), thrombocytopenia (n = 3), infections requiring hospitalization (n = 2), febrile neutropenia (n = 1), neutropenia (n = 1), skin rash (n = 1), diarrhea (n = 1), and elevated alkaline phosphatase (n = 1). Immune-related adverse events included hypothyroidism (n = 3), hyperthyroidism (n = 1), inflammatory polyarthritis (n = 1), and reactivation of latent tuberculosis (n = 1), all managed at Grade 2 severity. Delay in initiation of cycle 2 (5–7 days) occurred in 4 of 33 patients (12·1%) due to the time required for adverse events to resolve between cycles. Full adverse event data are presented in Table 2.

**Table 2:** Treatment related adverse effects during neoadjuvant therapy as per CTCAE.

Adverse event	Any grade	Grade 1/2	Grade 3	Grade 4
Nausea	7 (20·6%)	7 (20·6%)	–	–
Vomiting	7 (20·6%)	7 (20·6%)	–	–
Myalgia	5 (14·7%)	5 (14·7%)	–	–
Anemia	9 (26·5%)	6 (17·6%)	3 (8·8%)	–
Neutropenia	3 (8·8%)	2 (5·9%)	1 (2·9%)	–
Febrile Neutropenia	1 (2·9%)	–	1 (2·9%)	–
Infections	4 (11·8%)	2 (5·9%)	2 (5·9%)	–
Thrombocytopenia	6 (17·6%)	3 (8·8%)	3 (8·8%)	–
Skin rashes	27 (79·4%)	26 (76·5%)	1 (2·9%)	–
Raised creatinine	2 (5·9%)	1 (2·9%)	–	1 (2·9%)
Diarrhea	5 (14·7%)	3 (8·8%)	1 (2·9%)	1 (2·9%)
Fatigue	9 (26·5%)	9 (26·5%)	–	–
AST/ALT increased	2 (5·9%)	2 (5·9%)	–	–
Alkaline Phosphatase increased	–	–	1 (2·9%)	–
Bilirubin Increased	1 (2·9%)	1 (2·9%)	–	–
Oral mucositis	9 (26·5%)	9 (26·5%)	–	–
Immune-related Adverse events				
Hypothyroidism	3 (8·8%)	3 (8·8%)	–	–
Hyperthyroidism	1 (2·9%)	1 (2·9%)	–	–
Inflammatory polyarthritis	1 (2·9%)	1 (2·9%)	–	–
Infections (Reactivation of latent Tuberculosis)	1 (2·9%)	1 (2·9%)	–	–

Among surgical patients, significant postoperative complications (Clavien–Dindo Grade ≥ III) occurred in three patients: hypoxic respiratory failure with lung collapse (Grade IVA), secondary hemorrhage requiring intervention (Grade IIIA) and flap dehiscence with infection requiring multiple procedures (Grade IIIB) (Supplementary Table S3A).

There was no undue delay between surgery and adjuvant chemoradiation. All but one patient received adjuvant radiation therapy (RT), with doses of 60–66 Gy over 30–33 fractions. Of 30 patients receiving RT, all but one also received concurrent weekly cisplatin (median 3 cycles; IQR 3–4). One patient received RT alone due to ECOG PS 2; another received no adjuvant therapy due to prolonged postoperative complications. During RT, Grade 4 mucositis occurred in two patients and Grade 3 mucositis in four. Other Grade 3 events included febrile neutropenia (n = 2), radiation dermatitis, fatigue, and thrombocytopenia, yielding CTCAE Grade ≥3 toxicities in 26·6% (8/30) of patients receiving RT. All adverse events related to local therapy are listed in Supplementary Table S3B.

## Discussion

In this prospective phase II trial, the NeoLOCUS regimen combining carboplatin and nab-paclitaxel every three weeks with low-dose nivolumab and oral metronomic therapy yielded a 66% radiological response rate and a 90% overall R0 resection rate, with 75·7% of surgical conversions occurring after two cycles. These outcomes compare favorably to historical response and resectability rates of 30–45% with standard doublet and triplet NACT regimens.^10^^,^^12^, ^13^, ^14^^,^^17^^,^^21^ In borderline resectable OSCC, where curability hinges on achieving operability, these results carry important clinical implications.

The safety profile of our regimen further distinguishes it from standard TPF-based approaches. In Noronha and colleagues' phase III trial, 72·5% of patients receiving TPF experienced Grade ≥3 toxicities, including febrile neutropenia (25%), diarrhea (14%), anemia (14%), mucositis (11%), and two treatment-related deaths.^14^ Real-world data reinforce TPF's resource-intensive nature, with frequent hospitalizations and routine need for growth factor support.^21^ Notably, most TPF regimens reassess after a median of 3 cycles, which may delay transition to definitive local therapy and increase cumulative toxicities. By contrast, our regimen was delivered entirely in the outpatient setting, required no growth factor support, demonstrated manageable toxicities, and was not associated with any treatment-related deaths. Most patients required only a shorter 6-week course of treatment. These advantages in feasibility and safety underscore its potential as a practical neoadjuvant option.

Retrospective studies from India have explored similar combinations of platinum doublet chemotherapy with OMT in borderline resectable OSCC, but without immunotherapy. Kashyap and colleagues reported a resectability rate of 65% in a cohort of 14 patients treated with paclitaxel-carboplatin plus OMT, with partial responses in 65% and median progression-free survival of 11·4 months.^29^ Two larger retrospective series presented at ASCO 2022 reported resectability rates of 60–70% using similar regimens, with Grade ≥3 toxicities ranging from 28% to 36%.^37^^,^^38^ Compared with these cohorts, our study demonstrated higher surgical conversion rates, suggesting that adding low-dose nivolumab may enhance tumor regression and immune modulation. Nonetheless, differences in institutional definitions of borderline resectability warrant cautious interpretation.

NACT studies in locally advanced or borderline resectable OSCC have reported pCR rates between 8% and 13·4%.^21^^,^^39^ Our study showed an MPR of 41·4%, including 13·8% pCR. This multidrug regimen may promote more consistent, deeper tumor regression than NACT alone, highlighting potential synergy between cytotoxic therapy and immune modulation. pCR and MPR are recognized as surrogate markers for improved survival in borderline resectable OSCC and resectable HNSCC, respectively.^21^^,^^40^ In our study, EFS analysis stratified by pathological response showed a numerical trend toward better outcomes in MPR patients (median EFS 18·5 months) versus non-MPR (16·1 months), though the difference was not statistically significant, likely due to the modest sample size and short follow-up.

The NeoLOCUS regimen was designed to integrate OMT with low-dose immunotherapy and a platinum doublet backbone. Metronomic therapy modulates the tumor microenvironment through antiangiogenic effects, depletion of regulatory T cells (Tregs) and myeloid-derived suppressor cells, and enhancement of dendritic cell maturation and cytotoxic T-cell function. Methotrexate and celecoxib, key OMT components, have shown Treg-suppressive effects in preclinical models.^31^^,^^32^ Low-dose nivolumab sustains PD-1 receptor occupancy and immune activation, with proven efficacy in HNSCC.^27^^,^^28^^,^^33^^,^^34^ Supporting this mechanism, immune profiling in our trial showed a significant reduction in FOXP3^+^ Tregs among good responders (median Δ −1·0; p = 0·014) and a greater decrease compared to poor responders (p = 0·036). These exploratory findings are consistent with the hypothesis that combining low-dose PD-1 blockade with metronomic therapy may modulate the tumor immune microenvironment in association with deeper pathological responses. These observations should be regarded as hypothesis-generating, as the immune profiling analyses were conducted in a small, underpowered subset of patients and may have been influenced by unmeasured clinical and biological confounders. Larger, prospectively powered translational cohorts will be required to assess the independent association of FOXP3^+^ Tregs with pathological response and event-free survival.

Volumetric responses have been shown to be useful biomarkers for predicting outcomes in nasopharyngeal cancer, although they have not been widely evaluated in other head and neck sites.^41^^,^^42^ This may be particularly relevant in oral cavity tumors, where irregular morphology limits the reliability of unidimensional RECIST. Patil and colleagues demonstrated poor concordance between RECIST response and pathological outcomes after induction chemotherapy, with some patients achieving pCR despite minimal shrinkage.^43^ Kale and colleagues compared RECIST 1·1 with volumetric RECIST in locally advanced HNSCC and, by applying conventional RECIST thresholds directly to volumetric measurements, found major discordance because many tumors categorized as stable by RECIST were reclassified as partial responses.^44^ A phase II study of neoadjuvant nivolumab with or without ipilimumab in oral cavity cancer reported volumetric response rates of 50–53 percent using bidirectional measurements, underscoring the potential role of volumetric assessment.^45^ In our study, we applied a vPR threshold of 65 percent reduction, the volumetric analog of a 30 percent decrease in diameter, rather than the exact RECIST thresholds. This higher cut-off likely accounts for the lower vPR proportion compared with RECIST PR. However, the median volumetric decrease of −54·6 percent (IQR −74 to −33) explains why some patients were considered resectable despite being classified as SD/vSD by RECIST 1·1 or vRECIST (Supplemental Figure S2). Neither RECIST nor volumetric responses correlated with pathological response or survival. Larger oral cavity focused studies are needed to validate volumetric thresholds and their prognostic value.

This study has several limitations. The modest sample size and predominance of younger, fit patients limit generalizability. However, all eligible patients meeting inclusion criteria were prospectively enrolled to minimize selection bias. The definition of borderline resectability was determined by our institutional MDT and may not apply uniformly across centers; nonetheless, predefined consistent criteria reduced interobserver variability. The short follow-up limits assessment of long-term outcomes, but ongoing surveillance is capturing mature survival data. TiME profiling was added as a protocol amendment and was feasible only in a subset of patients. vRECIST did not contribute to outcome prediction, likely due to the small sample size and lack of validated volumetric cut-offs for oral cavity tumors.

This is a prospective study from the Indian population to report on a neoadjuvant regimen combining platinum doublet chemotherapy, oral metronomic therapy, and low-dose nivolumab in borderline resectable OSCC. The NeoLOCUS regimen was associated with encouraging rates of surgical conversion and pathological response, with a favorable safety profile. Its outpatient delivery without routine growth factor support suggests potential simplification of treatment delivery and resource advantages in appropriate settings. However, these findings need validation in larger, multicentre randomized studies to define the clinical impact and generalizability of this approach.

## Contributors

Conceptualization: PKM, AJT, BKS, KV, JRi, MA, MT, AJ, DBT, RI, JRn, AS Data Curation: PKM, CR, SS, AJT, BKS, JVW, KV, JRi, MA, MT, AJ, SKS, JTG, DBT, DSP, KH, AKS, MM, RCM, JR, JRn Formal Analysis: PKM, JRn, AJ, SKS, JTG, DBT, DSP, KH, AKS Funding Acquisition: PKM, AS Investigation: PKM, CR, SS, AOJ, AJT, BKS, JVW, KV, JRi, MA, MT, AJ, SKS, JTG, DBT, DSP, KH, AKS, MM, SP, RCM, RI Methodology: PKM, AOJ, AJ, SKS, JTG, DBT, DSP, KH, AKS, SP, RI, AS Project Administration: AJT, BKS, RI, AS Resources: CR, SS, AJT, BKS, JVW, KV, JRi, MA, MT, MM, RCM, SP, RI, JRn, AS Software: JRn, PKM Supervision: AJT, BKS, RCM, RI, AS Validation: PKM, CR, SS, AOJ, AJ, SKS, JTG, DBT, DSP, KH, AKS, JRn Visualization: PKM, CR, SS, JRn Writing—Original Draft: PKM, AJ, SKS, JTG, DBT, DSP, KH, AKS Writing—Review & Editing: All authors.

All authors reviewed and approved the final version of the manuscript.

## Data sharing statement

Deidentified individual participant data and a data dictionary will be available beginning with publication, along with the study protocol and statistical analysis plan. The primary dataset is included in the article and Supplementary Files. Additional data may be requested from the corresponding author for research purposes, subject to approval of a proposal and a signed data access agreement.

## Declaration of interests

All authors declare no conflict of interest.

## Figures and Tables

**SF1A:**
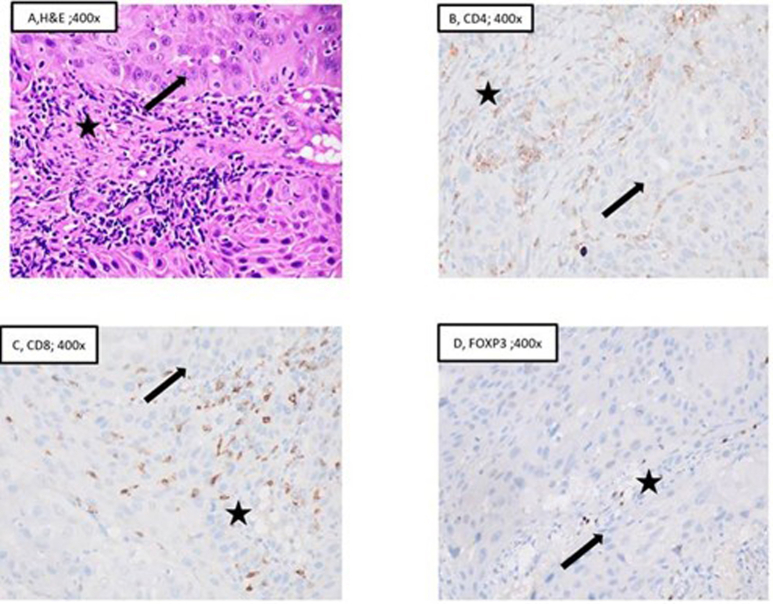
Supplemental Figure SF1A. Baseline tumor histology and immune cell infiltration (small biopsy specimen). Representative baseline small biopsy specimen of oral cavity squamous cell carcinoma demonstrating tumor histology and stromal tumor-infiltrating lymphocytes (sTILs). Carcinoma cells are indicated by arrows and stromal TILs by stars. (A) Hematoxylin and eosin (H&E) staining shows moderately differentiated squamous cell carcinoma with stromal lymphocytic infiltration; average sTILs 20%. (B) CD4 immunohistochemistry highlights stromal CD4+ T lymphocytes; average sTILs 25%. (C) CD8 immunohistochemistry highlights stromal CD8+ T lymphocytes; average sTILs 10%. (D) FOXP3 immunohistochemistry highlights regulatory T cells; average sTILs 1%. All immunohistochemistry images shown at ×400 magnification.

**SF1B:**
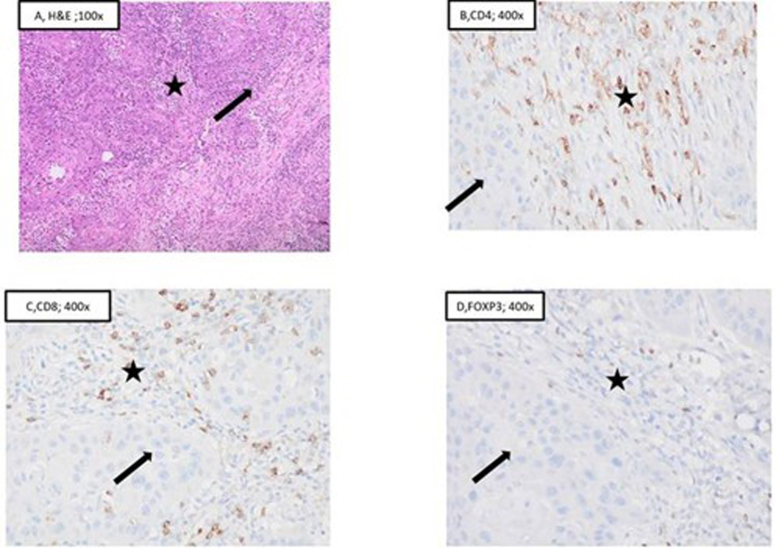
Supplemental Figure SF1B. Post-neoadjuvant tumor histology and immune cell infiltration (surgical specimen). Representative post-neoadjuvant surgical specimen of oral cavity squamous cell carcinoma demonstrating tumor histology and stromal tumor-infiltrating lymphocytes (sTILs). Carcinoma cells are indicated by arrows and stromal TILs by stars. (A) Hematoxylin and eosin (H&E) staining shows moderately differentiated squamous cell carcinoma with stromal lymphocytic infiltration; average sTILs 15%. (B) CD4 immunohistochemistry highlights stromal CD4+ T lymphocytes; average sTILs 20%. (C) CD8 immunohistochemistry highlights stromal CD8+ T lymphocytes; average sTILs 10%. (D) FOXP3 immunohistochemistry highlights regulatory T cells; average sTILs 1%. All immunohistochemistry images shown at ×400 magnification.

**SF2:**
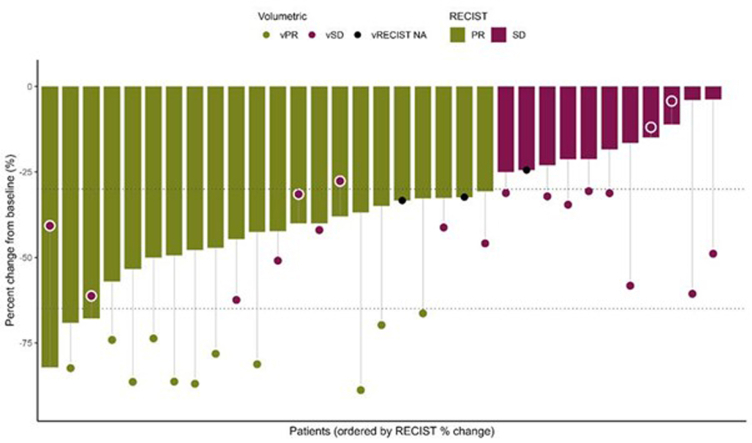
Supplemental Figure SF2. Radiological Tumor Response by RECIST With Integrated Volumetric Assessment. Waterfall plot depicting radiological tumor response following neoadjuvant therapy. Bars represent percentage change in tumor size by RECIST 1.1, with olive green indicating partial response (PR) and maroon indicating stable disease (SD). Overlaid dots represent volumetric response categories, including volumetric partial response (vPR) and volumetric stable disease (vSD). Black dots indicate missing volumetric data (NA). Horizontal dashed lines denote the RECIST partial response threshold (−30%) and the predefined volumetric partial response threshold (−65%). Patients are ordered from left to right by RECIST percentage change.

## Appendix A Supplementary data

Supplementary Tables

NeoLOCUS Protocol
