# Supplementary material for: Low-dose nivolumab with neoadjuvant chemotherapy and oral metronomic therapy in borderline resectable oral cavity squamous cell carcinoma: a phase II trial
Source: Lancet Reg Health Southeast Asia. 2026 Mar 5;47:100743. doi: 10.1016/j.lansea.2026.100743 (PMC12969808; doi:10.1016/j.lansea.2026.100743)
Supplement: Supplementary Tables [file mmc1.docx]

***Supplemental Table* 1 : Pathological response – Overall, Primary site and Nodal site**

| Pathological response | MPR:pCR | MPR:Non-pCR | Non-MPR |
| --- | --- | --- | --- |
| Overall | 4 | 8 | 17 |
| Primary site | 7 | 10 | 12 |
| Neck nodes | 10 | 5 | 11 |

***Supplementary Table ST2A* : Baseline Immune Biomarker (n=27)**

| **Marker** | **Mean** | **Median** | **IQR** |
| --- | --- | --- | --- |
| sTILs (%) | 14 | 10·0 | 10·0–15·0 |
| CD4⁺ T cells | 10·5 | 10·0 | 5·0–10·0 |
| CD8⁺ T cells | 6·5 | 5·0 | 5·0–10·0 |
| FOXP3⁺ Tregs | 2·0 | 1·0 | 1·0–1·0 |
| CD8/FOXP3 Ratio | 5·2 | 5·0 | 2·0–5·0 |

***Supplemental Table ST2B*: Post-treatment Immune Biomarkers (n=20)**

| **Marker** | **Mean** | **Median** | **IQR** |
| --- | --- | --- | --- |
| sTILs (%) | 17 | 15·0 | 10·0–20·0 |
| CD4⁺ T cells | 15·5 | 10 | 10·0–20·0 |
| CD8⁺ T cells | 8·5 | 10 | 5·0–10·0 |
| FOXP3⁺ Tregs | 1·0 | 1·0 | 0·5–1·0 |
| CD8/FOXP3 Ratio | 7·9 | 5·0 | 5·0–10·0 |

***Supplementary Table* ST3A: Postoperative Surgical Complications (Clavien-Dindo Grades)**

| **Adverse Event** | **Any Grade** | **Grade II** | **Grade III** | **Grade IV** |
| --- | --- | --- | --- | --- |
| Flap congestion needing debridement | 1 | 1 | 0 | 0 |
| Flap dehiscence with SSI and multiple flaps | 1 | 0 | 1 | 0 |
| Flap edge dehiscence | 1 | 1 | 0 | 0 |
| Flap site bleeding | 1 | 0 | 1 | 0 |
| Flap site necrosis needing debridement | 1 | 1 | 0 | 0 |
| Lung collapse with hypoxic respiratory  failure | 1 | 0 | 0 | 1 |

***Supplementary Table* ST3B: CTCAE Grading of Toxicities During Adjuvant Chemoradiation**

| **Adverse Event** | **Any Grade** | **Grade 1/2** | **Grade 3** | **Grade 4** |
| --- | --- | --- | --- | --- |
| Anemia | 3 | 3 | 0 | 0 |
| Dermatitis | 29 | 27 | 2 | 0 |
| Fatigue | 20 | 19 | 1 | 0 |
| Febrile neutropenia | 2 | - | 2 | 0 |
| Mucositis | 29 | 26 | 1 | 2 |
| Neutropenia | 4 | 4 | 0 | 0 |
| Thrombocytopenia | 3 | 3 | 0 | 0 |

***Supplemental Table 4 -* Volumetric data**

| **Total Tumor Volume (TTV)** | **Median** | **IQR** |
| --- | --- | --- |
| Baseline TTV | 35·85 | 28·3-52·7 |
| Reassessment TTV | 17·1 | 8·6-25·2 |
| %∆TTV  (Reassessment vs. Baseline) | -54·59 | (-74) – (-32·7) |
