# Supplementary material for: Low-dose nivolumab with neoadjuvant chemotherapy and oral metronomic therapy in borderline resectable oral cavity squamous cell carcinoma: a phase II trial
Source: Lancet Reg Health Southeast Asia. 2026 Mar 5;47:100743. doi: 10.1016/j.lansea.2026.100743 (PMC12969808; doi:10.1016/j.lansea.2026.100743)
Supplement: NeoLOCUS Protocol [file mmc2.docx]

1. **ADMINISTRATIVE INFORMATION**

**Protocol Title**: *Neoadjuvant Low-Dose Nivolumab with Carboplatin, Nab-Paclitaxel, Erlotinib, Methotrexate and Celecoxib in Borderline Resectable Oral Cavity Squamous Cell Carcinoma* (NeoLOCUS)

**Trial Registration**: Clinical Trial Registry of India CTRI/2023/04/051617

**Ethics Approval**: IRB Min No. 15148 dated 25.01.2023 (Christian Medical College, Vellore)

**Funding Source**: Fluid Research Grant, Christian Medical College, Vellore

**Sponsor**: Christian Medical College, Vellore

**Principal Investigator**: Dr Praveen Kumar Marimuthu

[drmpk91@gmail.com](mailto:drmpk91@gmail.com)

1. **BACKGROUND AND RATIONALE (Layman version)**

A fair proportion of Locally Advanced Oral cavity Squamous cell carcinomas patients cannot undergo upfront surgery because of the extent of tumor. For these patients, options are Upfront chemotherapy along with radiation or Initial chemotherapy followed by reassessment for operability. Several studies have demonstrated inferior outcomes with the non-surgical option in oral cavity cancers. Therefore, the second option seems valid for these patients. Majority of patients in Indian studies have received several regimens like Cisplatin and 5-Fluorouracil, Cisplatin and Docetaxel, Cisplatin and Paclitaxel, Carboplatin and Paclitaxel with or without low dose oral chemotherapy, Carboplatin and Docetaxel and less commonly, the three-drug regimen Docetaxel, Cisplatin and 5-Fluorouracil. This study attempts to answer the optimal treatment regimen for use in the Upfront setting. Amongst the various regimen, the combination of carboplatin, paclitaxel and oral low dose chemotherapy was reported to have favorable outcomes while having an acceptable toxicity profile in a recently presented study from Tata Memorial Hospital, Mumbai. In our study, we add a low dose immunotherapy drug to this combination and assess the outcomes. The primary outcome parameter is the percentage of patients who can undergo surgery without leaving any residual tumor behind after initial treatment with the study regimen. The secondary objectives of the study will be to assess the change in size and extent of the tumor with the help of a CT scan, change in the tumor assessed by pathologist after surgery, time that passes after the treatment completion before patient develops a recurrence, proportion of patients alive at 2 years after the treatment, proportion of patients completing planned cycles of chemoimmunotherapy, Incidence of laboratory and clinical adverse events and Quality of life at several time points before, during and after treatment.

BACKGROUND AND RATIONALE (SCIENTIFIC VERSION):

**Epidemiology of Head and Neck cancers in the Indian Population**

In India, Cancer of the mouth and oral tongue has the highest incidence rate (13.9 per 100000 person years) amongst all cancers. In Chennai, the incidence rate of Mouth cancer in males and females are 7.1 and 3.8 respectively. Meanwhile, the incidence rate of Oral tongue cancer in males and females in Chennai are 4.5 and 1.6 respectively.^(1)^ The National cancer registry programme had reported a projected incidence of oral cancer in 1,84,838 people in the year of 2020.^2^ Oral cavity cancers are the most common cause of cancer related mortality in India among men (7.7 per 100000 person years).^(3)^ The higher incidence rates is related to the prevalent use of oral tobacco, tobacco smoking and alcohol.^(4,5)^

**Overview of the problem**

- Squamous cell carcinoma is the most common histology reported in Oral cavity cancers.^(6)^ Majority of oral cavity squamous cell carcinomas are locally advanced at the time of initial presentation with 5-year survival of only 50-60%.^(7)^ Ipsilateral neck node involvement is the most significant predictor of survival in these group of patients. Locally advanced oral cavity cancer has high rates of locoregional recurrence (up to 35%) and distant metastasis (up to 15%) after initial treatment. ^(8)^ A fair proportion of these cases, when deemed unresectable, are treated with non-surgical option of External Beam radiotherapy with or without concurrent chemotherapy either upfront or after a trial of induction chemotherapy. Several studies in literature have shown inferior outcomes with the non-surgical approach in oral cavity cancer. ^(9-11,15)^ The best choice of induction chemotherapy in terms of favorable outcomes as well as acceptable toxicity profile, particularly in the Indian population is under investigation. Such an approach can not only improve resectability but also might help in reducing the risk of distant disease recurrence apart from enabling organ preservation. This forms the basis for this research.

**Studies on Neoadjuvant chemotherapy using Docetaxel + Cisplatin regimen**

In a large retrospective series reported by Patil et al, patients with very locally advanced technically unresectable oral cavity cancers were administered induction chemotherapy with either 3-drug (10.2%- 74 patients) or 2-drug (89.8%-647 patients) regimen. 245 patients received docetaxel and cisplatin chemotherapy. 40.34% of patients who received 2-drug chemotherapy regimen achieved resectability. The response rate with the docetaxel based 2-drug regimen was 30.4%. The locoregional control and OS was superior in patients undergoing surgery as opposed to those who did not. ^(9)^

In another retrospective series, Patil et al reported the resectability rates following 3-drug versus 2-drug regimen of induction chemotherapy in locally advanced technically unresectable oral cavity cancers. ^(10)^ 68% of patients who received 3-drug regime achieved resectability compared to 37% who received 2-drug regimen. Incidence of febrile neutropenia was 34.6% in 3-drug regimen vs 3% in 2-drug regimen. The authors concluded that patients who achieve resectability following induction chemotherapy derive an overall survival benefit.

Malik et al evaluated the role of NACT in patients with HNSCC with unresectable cervical nodes. Majority of the study population (67.2%) had oral cavity as primary site. Majority (59%) patients received docetaxel and cisplatin. 65% patients had favorable nodal response to NACT and 21.5% of patients underwent curative surgery leading to improved OS.^(11)^ In a retrospective analysis of prospectively collected data, Noronha et al compared outcomes of induction chemotherapy using 3-drug vs 2-drug regimen for locally advanced oral cavity cancers.^(12)^ In the 2-drug regimen arm, majority of the patients received Docetaxel + cisplatin (nearly 40%). The primary outcome variable was radiological response rate which was 30.3% for the 2-drug regime compared to 50% for the 3-drug regime. However, the 3-drug regime was used in only 9% of the patient population.

There were many challenges in administering the 3-drug regime as routine for this patient population. This regimen requires 5 days continuous infusion of 5FU, extensive monitoring, prophylactic granulocyte-colony stimulating factor, prophylactic antibiotics, intensive supportive care, logistical issues of unavailability of indoor beds for admission, long travelling distance between place of stay and hospital and financial constraints.

Joshi et al reported the outcomes following induction chemotherapy in T4b oral cavity cancers.^(13)^ Majority (80%) of these patients received 2-drug chemotherapy regimen of which 19% (21 patients) received docetaxel and cisplatin. Regimen-specific resectability rates were not reported but resectability was achieved in 25% of the patients who received 2-drug regimen.

In an audit of patients receiving 3-drug chemotherapy regimen for LA-OCSCC, Patil et al had reported Grade 3 or higher toxicities occurring in 91.67% of the patients.^(14)^

The notable Grade 4 toxicities included Febrile neutropenia, thrombocytopenia, dyselectrolytemia, mucositis and diarrhea occurring in one-third of treated patients.

A retrospective audit was recently published by Mishra et al evaluating induction chemotherapy using either 3-drug DCF (26 patients) or 2-drug chemotherapy regimen (88 patients) in locally advanced unresectable HNSCC. Out of 88 patients receiving doublet chemotherapy regimen, 42 (47.7%) received DC protocol, 30 (34.1%) received 3-weekly PC and 16 (18.2%) received weekly PC regimen. Resectability was achieved in 32.3% of three-drug regimen patients and 26.1% of other patients. Response assessment showed partial response in 41.9% of patients with three-drug regimens and 37.5% of patients with other regimens. The estimated median survival for patients who could undergo surgery was 18 months compared to nonsurgical patients having median OS 9 months.^15^

**Table 1: Studies Using Docetaxel + Cisplatin (DC) as NACT in LA-HNSCC**

| Author,  Year of Publication | No. of patients receiving DC/sample size | Study population | Resectability rate  Post induction | ORR | Toxicities (all patients) | OS (all patients) |
| --- | --- | --- | --- | --- | --- | --- |
| Patil et al, 2014 | 245/721 | Very locally advanced Technically unresectable OCSCC | 40.3% | RR 30% | NR | 2-year OS:  47% (Resection) vs 20% (No resection) |
| Patil et al, 2013 | 17/95 | Locally advanced Technically unresectable OCSCC | 37.9% | PR 27%  SD 59%  PD 14% | Grade 3/4 toxicities: FN 3%, CINV 5%, CID 2% | mOS 12.7 months (Resection) vs 8 months (No resection) |
| Malik et al, 2019 | 30/51 | LAHNSCC with unresectable cervical nodes | 21.5% | CR 4%  PR 61%  SD 14%  PD 21% | NR | mOS 24 months (Resection) vs 13 months (CTRT) |
| Noronha et al, 2015 | 97/245 | LAOCSCC | NR | RR 30% | NR | NR |
| Joshi et al, 2013 | 21/110 | T4b OCSCC | 25% | PR 28%  SD 49%  PD 23% | Grade 3/4 hematological toxicity rate 4.5% | mOS 18 months (Resection) vs 6.5 months (No resection) |
| Mishra et al*, 2022 | 42/88 | LAHNSCC | 26.1% | PR 37%  SD 40%  PD 23% | Grade 3/4 Anemia 26.4%,  Grade 3/4 Thrombocytopenia 5.7%,  Grade 3/4 CINV 3.4%,  Grade 3/4 Diarrhea 4.6%  Grade 3/4 cardiac event 1.1% | mOS 18 months (Resection) vs 9 months (No resection) |

*****Outcomes and toxicities not reported specifically for docetaxel and cisplatin

NR-Not Reported; RR- Response Rate; ORR- Objective Response Rate; CR-Complete Response

SD-Stable Disease; PR-Partial Response; PD-Progressive Disease; CTRT- Concurrent chemoradiotherapy; CINV- Chemotherapy induced Nausea and Vomiting; CID- Chemotherapy induced Diarrhea; FN- Febrile Neutropenia

**Studies on Neoadjuvant chemotherapy using Carboplatin + Paclitaxel regimen +/- OMCT**

Pathak et al reported the outcomes of NACT with paclitaxel plus carboplatin with triple OMCT using erlotinib, methotrexate and celecoxib in locally advanced borderline resectable/ technically unresectable head and neck cancers in a retrospective analysis of 72 patients. Paclitaxel and carboplatin were given either weekly or three-weekly along with once daily erlotinib, twice daily celecoxib and once weekly methotrexate. 34 patients among 40 borderline resectable underwent surgery. PR was observed in 61.1%, SD in 26.4%, PD in 11.1%. Median PFS was not reached and 2-year PFS was 52.9%. Median OS was 18.8 months and 2 – year OS was 45.7%.^16^

In a retrospective analysis of prospectively maintained data, Singh et al analyzed outcomes following metronomic systemic PCm (Paclitaxel, carboplatin + metronomic) NACT in head and neck cancer. After 6 cycles of weekly schedule, 52 patients were assessed for clinical and radiological outcomes. 94.6% patients had oral cavity primaries. PR was seen in 96.4% patients, 3.6% patients had SD and none had PD. 84% of technically unresectable patients were deemed resectable of which 62.5% underwent surgery. Pathological complete response was seen in 48.2% patients. Grade 3/4 oral mucositis was seen in 10.7% patients , diarrhea in 7.1% and FN in 1.7% patients.^17^

Kashyap et al studied the safety and efficacy of carboplatin + paclitaxel along with OMCT in patients with technically unresectable OCSCC in 14 patients. After a median of 3 cycles of NACT with this regimen, the authors reported an impressive resectability rate of 65%. Nine patients (65%) had PR and none had PD. Grade 3 Febrile Neutropenia was observed in 14% patients and grade 3 anemia in 7% patients. Median PFS was 11.4 months and OS at 15 months was 63.5%. Apart from the encouraging outcomes, this regimen was also less resource intensive and well tolerated in the patient population studied. ^18^

Rudresha et al retrospectively analysed the outcomes of induction chemotherapy in Locally advanced T4b OCSCC using either a 3-drug DCF regimen (13 patients-11.2%) or 2-drug carboplatin and paclitaxel regimen (103 patients-88.8%). ^19^ Objective response rate was the primary outcome of the analysis. The authors reported PR in 20 patients (17.3%), SD in 68 patients (58.6%), progressive disease in 28 patients (24.1%). Resectability was achieved in 20 patients (17.3%). Those who underwent surgery had a significantly better median OS compared to those who received non-surgical treatment.

Herman et al compared carboplatin and paclitaxel induction chemotherapy to 3-drug regimen of DCF for LAHNSCC prior to CTRT. Interestingly, they reported better outcomes with the 2-drug regimen in terms of superior 1-year locoregional control rates (80.5% vs 55.5%). PFS was in favour of 2-drug regimen 73.2% vs 60.7%. Tolerability was better for the 2-drug regimen with lesser chances of renal toxicity compared to DCF. In this study, 23 oral cavity cancer patients (25.6% of total patient population) had received the carboplatin and paclitaxel regimen of chemotherapy.^20^

The previously discussed retrospective series including the largest study by Patil et al comprised of 51 patients who received a combination of carboplatin and paclitaxel. For patients with unresectable cervical nodes as in the Malik et al study, nine patients had received carboplatin + paclitaxel regimen as NACT. The outcomes of these studies have been explained earlier.

**Table 2: Studies using Carboplatin + paclitaxel +/- OMCT as NACT in LAHNSCC**

| Author, Year of Publication | Number of patients receiving PC/sample size | Study population | Resectability rate  Post induction | ORR | Toxicities | PFS/OS  (All patients) |
| --- | --- | --- | --- | --- | --- | --- |
| Patil et al, 2014 | 51/721 | Very locally advanced Technically unresectable OCSCC | 40.3% | RR30% | NR | 2-year OS:  47% (Resection) vs 20% (No resection) |
| Patil et al, 2013 | 10/95 | Locally advanced Technically unresectable OCSCC | 37.9% | PR 27%  SD 59%  PD 14% | Grade 3/4 toxicities: FN 3%, CINV 5%, CID 2% | mOS 12.7 months (Resection) vs 8 months (No resection) |
| Malik et al, 2019 | 30/51 | LAHNSCC with unresectable cervical nodes | 21.5% | CR 4%  PR 61%  SD 14%  PD 21% | NR | mOS 24 months (Resection) vs 13 months (CTRT) |
| Kashyap et al*, 2021 | 14/14 | Technically unresectable OCSCC | 65% | 65% PR 0%PD | Grade 3/4 FN 14%  Grade 3 Anemia 7% | OS at 15 months 63.5% |
| Pathak et al*, 2022 | 72/72 | Locally Advanced Borderline Resectable/ Technically Unresectable HNSCC | NR | 44% PR 19% SD  11% PD  NA1.4% | NR | 2 Year PFS 53%, mOS 18.8 months, 2 year OS 45.7% |
| Singh et al*, 2022 | 52/52 | Locally Advanced Borderline Resectable/ Technically Unresectable HNSCC | 84 % (62.5% underwent surgery) | PR96%, SD 4% | Grade 3/4 Toxicities: oral mucositis 10.7% , diarrhea 7.1%, FN 1.7% | mPFS 5.23 months non-surgery group, not reached in other groups, mOS not reached |
| Rudresha et al, 2017 | 103/116 | T4b OCSCC | 17.3% | PR 17%  SD 59% PD 24% | Grade 3/4 hematological toxicities:  21.3% | mOS 19.7 months (Resection) vs 7.1 months (No resection) |
| Mishra et al €, 2022 | 46/88 | LAHNSCC | 26.1% | PR 37%  SD 40%  PD 23% | Grade 3/4 Anemia 26.4%,  Grade 3/4 Thrombocytopenia 5.7%,  Grade 3/4 CINV 3.4%,  Grade 3/4 Diarrhea 4.6%  Grade 3/4 cardiac event 1.1% | mOS 18 months (Resection) vs 9 months (No resection) |

*Studies using the carboplatin + paclitaxel+ triple OMCT combination

€ Outcomes and Toxicities reported are not specific to carboplatin + paclitaxel

ORR % were rounded off to the nearest value

RR- Response Rate; ORR- Objective Response Rate; CR-Complete Response; SD-Stable Disease; PR-Partial Response; PD-Progressive Disease; CTRT- Concurrent chemoradiotherapy; CINV- Chemotherapy induced Nausea and Vomiting; CID- Chemotherapy induced Diarrhea; FN- Febrile Neutropenia; PFS- Progression free survival; mOS- Median Overall Survival; mPFS- Median Progression Free survival

**Study rationale – Why use carboplatin + paclitaxel + OMCT + Low Dose Immunotherapy instead of Standard Docetaxel and Cisplatin?**

The combination of carboplatin, paclitaxel, erlotinib, methotrexate and celecoxib regimen had an impressive resectability rate ranging from 62-84% in a recently published study by TMH group, which is higher than any 2-drug (40%) or 3-drug (50%) regimen reported in literature so far with acceptable toxicity. As discussed earlier, higher overall survival was observed across several studies for patients who undergo surgery when compared to those who do not. The toxicity profile of the study regime is more favorable as it does not have several side effects that are seen with standard Docetaxel + Cisplatin. These include nephrotoxicity, ototoxicity, severe vomiting. Use of study regimen in the neoadjuvant setting enables cisplatin to be used in the concurrent setting with RT, should the tumor remain unresectable post induction chemotherapy. The best use of cisplatin is in the concurrent setting in head and neck squamous cell carcinomas as a radiosensitizer with External Beam radiotherapy. Patients who received cisplatin in the induction phase may not tolerate as well when continued to use in the concurrent setting, should it be required. Also, the study regime can be used in all patient groups including cisplatin-ineligible individuals. The ORR of this 5-drug combination was 63% as reported by TMH group. Our study attempts to find if adding low dose nivolumab to this combination further increases the response rate. Low dose nivolumab has already been studied in the palliative treatment of head and neck cancers both in the recurrent and newly diagnosed setting and found to be associated with improved response rates as well as overall survival in these group of patients.^21^

**Definition of Borderline Resectability criteria:**

The criteria were selected in our multidisciplinary clinic including head and neck surgeons, radiologists, medical oncologists and radiation oncologists.

1. Buccal mucosa primary with diffuse margins and peritumoral extension (edema) going up to the level of sigmoid notch of mandible but not above
2. Oral tongue primary with extension to posterior one-third of tongue or floor of mouth
3. Oral tongue primary with the tumor extending up to level of hyoid bone
4. Oral cavity tumor of any subsite with multiple skin nodules

REFERENCES

1. Miranda-Filho A, Bray F. Global patterns and trends in cancers of the lip, tongue and mouth. Oral oncology. 2020 Mar 1;102:104551.

2. Mathur P, Sathishkumar K, Chaturvedi M, Das P, Sudarshan KL, Santhappan S, Nallasamy V, John A, Narasimhan S, Roselind FS, ICMR-NCDIR-NCRP Investigator Group. Cancer statistics, 2020: report from national cancer registry programme, India. JCO Global oncology. 2020 Jul;6:1063-75.

3. Sung H, Ferlay J, Siegel RL, Laversanne M, Soerjomataram I, Jemal A, Bray F. Global cancer statistics 2020: GLOBOCAN estimates of incidence and mortality worldwide for 36 cancers in 185 countries. CA: a cancer journal for clinicians. 2021 May;71(3):209-49.

4. Sankaranarayanan R, Duffy SW, Day NE, Nair MK, Padmakumary G. A case‐control investigation of cancer of the oral tongue and the floor of the mouth in Southern India. International Journal of Cancer. 1989 Oct 15;44(4):617-21.

5. Muwonge R, Ramadas K, Sankila R, Thara S, Thomas G, Vinoda J, Sankaranarayanan R. Role of tobacco smoking, chewing and alcohol drinking in the risk of oral cancer in Trivandrum, India: a nested case-control design using incident cancer cases. Oral oncology. 2008 May 1;44(5):446-54.

6. Thompson LD. World Health Organization classification of tumours: pathology and genetics of head and neck tumours. Ear, Nose and Throat Journal. 2006 Feb 1;85(2):74-5.

7. Sankaranarayanan R. Oral cancer in India: an epidemiologic and clinical review. Oral surgery, oral medicine, oral pathology. 1990 Mar 1;69(3):325-30.

8. Thavarool SB, Muttath G, Nayanar S, Duraisamy K, Bhat P, Shringarpure K, Nayak P, Tripathy JP, Thaddeus A, Philip S. Improved survival among oral cancer patients: findings from a retrospective study at a tertiary care cancer centre in rural Kerala, India. World Journal of Surgical Oncology. 2019 Dec;17(1):1-7.

9. Patil VM, Prabhash K, Noronha V, Joshi A, Muddu V, Dhumal S, Arya S, Juvekar S, Chaturvedi P, Chaukar D, Pai P. Neoadjuvant chemotherapy followed by surgery in very locally advanced technically unresectable oral cavity cancers. Oral oncology. 2014 Oct 1;50(10):1000-4.

10. Patil VM, Noronha V, Muddu VK, Gulia S, Bhosale B, Arya S, Juvekar S, Chatturvedi P, Chaukar DA, Pai P, D'cruz A. Induction chemotherapy in technically unresectable locally advanced oral cavity cancers: does it make a difference? Indian Journal of Cancer. 2013 Jan 1;50(1):1

11. Malik A, Qayyumi BN, Mair M, Singhavi H, Mathur Y, Nair D, Ghosh-Laskar S, Agrawal JP, Prabash K, Chaturvedi P. Outcome of patients following neo-adjuvant chemotherapy for unresectable cervical nodes in head and neck squamous cell carcinomas. European Archives of Oto-Rhino-Laryngology. 2019 Feb;276(2):567-74

12. Noronha V, Patil V, Joshi A, Muddu V, Bhattacharjee A, Juvekar S, Arya S, Chaturvedi P, Chaukar D, Pai PS, Dcruz AK. Is taxane/platinum/5 fluorouracil superior to taxane/platinum alone and does docetaxel trump paclitaxel in induction therapy for locally advanced oral cavity cancers? Indian Journal of Cancer. 2015 Jan 1;52(1):70.

13. Joshi A, Patil VM, Noronha V, Juvekar S, Deshmukh A, Chatturvedi P, Chaukar DA, Agarwal JP, Ghosh S, Murthy V, D'cruz A. Is there a role of induction chemotherapy followed by resection in T4b oral cavity cancers? Indian Journal of Cancer. 2013 Oct 1;50(4):349.

14. Patil VM, Chakraborty S, Shenoy PK, Manuprasad A, Babu TS, Shivkumar T, Babu S, Bhatterjee A, Balasubramanian S. Tolerance and toxicity of neoadjuvant docetaxel, cisplatin and 5 fluorouracil regimen in technically unresectable oral cancer in resource limited rural based tertiary cancer center. Indian Journal of Cancer. 2014 Jan 1;51(1):69

15. Mishra BK, Gupta A, Kapoor A. Neoadjuvant chemotherapy in technically unresectable head and neck cancers: A retrospective audit [Internet]. ecancer. 2022 [cited 2022Nov6]. Available from: <https://ecancer.org/en/journal/article/1460-neoadjuvant-chemotherapy-in-technically-unresectable-head-and-neck-cancers-a-retrospective-audit>

16. Pathak S, Peelay Z, Patil VM, Noronha V, Menon NS, Chaturvedi P, Prabhash K. A retrospective analysis of patients administered neoadjuvant chemotherapy (NACT) with paclitaxel plus carboplatin with oral metronomic chemotherapy (OMCT) in locally advanced borderline resectable/technically unresectable head and neck cancers.

17. Singh GK, Prabhash K, Patil VM, Noronha V, Joshi A, Menon NS, Singh P, Qayyumi B, Singh R. Metronomic systemic PCm (paclitaxel, carboplatin+ metronomic) neoadjuvant chemotherapy in head and neck cancer.

18. Kashyap L, Patil V, Noronha V, Joshi A, Menon N, Jobanputra K, Saha S, Chaturvedi P, Banavali SD, Prabhash K. Efficacy and safety of neoadjuvant chemotherapy (NACT) with paclitaxel plus carboplatin and oral metronomic chemotherapy (OMCT) in patients with technically unresectable oral squamous cell carcinoma (OSCC). ecancermedicalscience. 2021;15.

19. Rudresha AH, Chaudhuri T, Lakshmaiah KC, Babu KG, Dasappa L, Jacob LA, Babu MS, Lokesh KN, Rajeev LK. Induction chemotherapy in locally advanced T4b oral cavity squamous cell cancers: A regional cancer center experience. Indian Journal of Cancer. 2017 Jan 1;54(1):35.

20. Herman LC, Chen L, Garnett A, Feldman LE, Smith B, Weichselbaum RR, Spiotto MT. Comparison of carboplatin–paclitaxel to docetaxel–cisplatin-5–flurouracil induction chemotherapy followed by concurrent chemoradiation for locally advanced head and neck cancer. Oral oncology. 2014 Jan 1;50(1):52-8.

21. Patil VM, Noronha V, Menon NS, Bhattacharjee A, Kumar S, Purandare N, Agrawal A, Puranik A, Nawale KP, Jogdhankar S, Alone M. Phase 3 randomised study evaluating the addition of low-dose nivolumab to palliative chemotherapy in head and neck cancer.

22. Fleming TR. One-sample multiple testing procedure for phase II clinical trials. Biometrics. 1982 Mar 1:143-51.

1. **PATIENT POPULATION:**

Adult patients who had biopsy-proven OSCC with an ECOG performance status of 0–1 and  deemed borderline resectable based on predefined criteria established by our Head and Neck Multidisciplinary Tumor Board (HN-MDT) were screened for enrolment. The criteria for borderline resectability included - a. Buccal mucosa primary with diffuse margins and peritumoral extension (edema) reaching up to the level of the sigmoid notch of the mandible but not above. b. Oral tongue primary with extension to the posterior one-third of the tongue, with peritumoral edema extending up to the hyoid bone. c. Oral cavity tumors of any subsite with extensive skin involvement confined to the surgical site.

**KEY CRITERIA**

*Inclusion Criteria*

Eligible patients met the following criteria:

- Adults >18 years of age with an ECOG performance status of 0–1.
- Biopsy-proven squamous cell carcinoma of the oral cavity.
- Clinical stage III to IVB disease as per AJCC 8th edition.
- Classified as borderline resectable by the HN-MDT.
- Provided written informed consent (participant or legal representative).
- Adequate baseline laboratory parameters:

o   Neutrophils ≥ 1,500/mm³

o   Platelets ≥ 100,000/mm³

o   Hemoglobin ≥ 8 g/dL

o   Estimated creatinine clearance ≥ 60 mL/min (Cockcroft-Gault formula)

*Exclusion Criteria*

Patients were excluded if they met any of the following:

1. Prior chemotherapy, radiotherapy, or surgery for the current oral cavity cancer.
2. Major surgery within 28 days before enrolment, or anticipated need for major surgery during the study (other than cancer-directed surgery).
3. Pregnant or breastfeeding individuals, or those unwilling to use contraception.
4. Documented baseline neuropathy of Common Terminology Criteria for Adverse Events (CTCAE) v5.0 grade ≥ 2.
5. Symptomatic congestive heart failure (NYHA class ≥ III).
6. Left ventricular ejection fraction <50% or other documented systolic dysfunction at baseline.
7. **INTERVENTION**

All patients received neoadjuvant chemo-immunotherapy consisting of:  Intravenous Carboplatin AUC5 Day 1+ Intravenous nab- paclitaxel 200 mg/m^2^ Day 1 with maximum dose capped at 300mg + Intravenous Nivolumab (Weight based dosing: <65 kg = 20 mg;  ≥65 kg = 40 mg)  Day 1 every 21 days for 2 cycles with OMT (Methotrexate 9 mg/m^2^ once weekly + Celecoxib 200mg twice daily + Erlotinib 100mg OD once daily) for 6 weeks. All patients had scheduled toxicity assessments on Day 10 and Day 20 of each cycle, with instructions to report intercurrent symptoms between visits.

INTERVENTION DOSE MODIFICATION

***Neoadjuvant Chemotherapy***

Full doses of all IV drugs will be given 3-weekly if neutrophil count >1.5 x 10^9^/L and platelets > 90 x 10^9^/L on the same day as chemotherapy or the previous day.

| Haematological Toxicity | Nab-Paclitaxel | Carboplatin |
| --- | --- | --- |
| No toxicity during cycle 1 | Full dose (200mg/m^2^) capped at 300mg | Full dose AUC 5 |
| On day of treatment ANC  500-1500 and/or platelets 50,000 - 90,000 | Proceed with full dose if counts recover within 3 days of planned date. Withhold all OMT meanwhile | Proceed with full dose if counts recover within 3 days of planned date. Withhold all OMT meanwhile |
| On day of treatment ANC <500 and platelets <50,000 | Delay cycle 2 by a week, proceed with 75% of dose after counts recovery (at next & all subsequent cycles if planned). Withhold all OMT, G-CSF use at physician’s discretion. | Delay cycle 2 by a week, proceed with AUC 4 after counts recovery (at next & all subsequent cycles if planned). Withhold all OMT, G-CSF use at physician’s discretion. |
| Febrile neutropenia  (≥ grade 3) | 75% of dose (at next & all  subsequent cycles if planned) | AUC 4 (at next & all subsequent cycles) |
| (If applicable)  Further occurrence of  ANC <500 or platelets ≤ 50000  despite dose reduction or  second occurrence of  febrile neutropenia | Discontinue further systemic therapy. Consult HN-MDT for further plan | Discontinue further systemic therapy. Consult HN-MDT for further plan |

***Oral Metronomic Therapy***

Full doses of all OMT drugs will be continued throughout 6 weeks if neutrophil count >1.5 x 10^9^/L and platelets > 90 x 10^9^/L at pre-specified timepoints.

| Haematological Toxicity | Action |
| --- | --- |
| On day of treatment ANC  500-1500 and/or platelets 50,000 - 90,000 | Withhold all OMT until counts recovery |
| On day of treatment ANC <500 and platelets <50,000 | Withhold all OMT until counts recovery |

| Non- Haematological Toxicity | Action |
| --- | --- |
| ***Erlotinib related Skin rash*** | |
| Grade 1–2 | Continue at full dose with supportive care (topical steroids, oral doxycycline). |
| Grade 3 | Consult dermatology. Interrupt erlotinib until resolution to ≤Grade 1. Resume at 50 mg daily. |
| Grade 4 | Discontinue erlotinib permanently. |
| ***Erlotinib-related Diarrhea*** | |
| Grade 1–2 | \|  \| Supportive care (loperamide, hydration). Continue at full dose. Consider withholding temporarily if grade 2 diarrhea persists for 2 or more days continuously until it resolves to grade 1 \| \| --- \| --- \| |
| Grade 3 | \|  \| Interrupt erlotinib until resolution to ≤Grade 1. Resume at 50 mg daily. \| \| --- \| --- \| |
| Grade 4 | \|  \| Discontinue erlotinib permanently. \| \| --- \| --- \| |
| ***Methotrexate-related Hepatotoxicity*** | |
| \| **Bilirubin >1.5 × ULN but ≤3 × ULN and/or ALT/AST >3 × ULN but ≤5 × ULN** \|  \| \| --- \| --- \| | \|  \| Withhold methotrexate until recovery to ≤1.5 × ULN for bilirubin and ≤3 × ULN for transaminases. On recovery, resume at 50% dose. \| \| --- \| --- \| |
| \| **Bilirubin >3 × ULN and/or ALT/AST >5 × ULN** \|  \| \| --- \| --- \| | \|  \| Discontinue methotrexate permanently. \| \| --- \| --- \| |
| \| **Recurrent hepatotoxicity despite dose reduction** \|  \| \| --- \| --- \| | \|  \| Discontinue methotrexate permanently and discuss in MDT for further plan. \| \| --- \| --- \| |
| ***Celecoxib related GI Event*** | |
| **Grade 1–2 (e.g., dyspepsia, mild gastritis)** | Continue celecoxib at full dose with supportive care. If acid suppression is required, prefer antacids or cautiously use H2 blockers ensuring appropriate separation from erlotinib dosing. Avoid proton pump inhibitors (PPIs) if possible due to potential interaction with immunotherapy and erlotinib. |
| **Grade 3 (e.g., severe gastritis, upper GI bleeding without hemodynamic instability)** | Withhold celecoxib. Resume at full dose upon resolution if no recurrence and after gastroenterology consultation. |
| **Grade 4 (e.g., GI perforation, massive bleeding requiring transfusion or surgery)** | Permanently discontinue celecoxib. Manage as per institutional GI emergency protocols. |
| ***OMT-Related Renal Dysfunction*** | |
| **Serum creatinine increase ≤1.5× baseline OR eGFR ≥60 mL/min/1.73m²** | Continue all OMT drugs at full dose. Monitor renal function weekly. |
| **Serum creatinine >1.5× baseline but ≤2× baseline OR eGFR 45–59 mL/min/1.73m²** | Withhold methotrexate and celecoxib. Continue erlotinib at full dose if no contraindications. Resume methotrexate and celecoxib at 50% dose when renal function recovers. Monitor renal function twice weekly. |
| **Serum creatinine >2× baseline OR eGFR <45 mL/min/1.73m²** | Withhold all OMT. Resume erlotinib, methotrexate at 50% dose when renal function recovers to eGFR ≥45 mL/min/1.73m². Celecoxib to be discontinued |
| **Persistent or progressive renal dysfunction despite dose modifications** | Permanently discontinue all OMT |

***Infusion Reactions***

A clinically significant Infusion reaction is one which meets the criteria for a CTCAE Grade 3 or 4 anaphylaxis. A patient may also discontinue induction chemotherapy if he/she has a hypersensitivity reaction which does not meet the criteria but which, in the opinion of the investigator, poses a significant risk on re-challenge. Patients who develop a Grade 3 or 4 hypersensitivity reaction to carboplatin may continue on nab-paclitaxel, OMT and LD-Nivo alone. For Grade 1 or 2 reaction to carboplatin, treatment may continue at the Investigator’s discretion with increased steroid, antihistamine and slower infusion time as per desensitization protocol of the department.

Caution should be exercised with cisplatin administration during the CRT phase of the treatment as additional premedication may be required due to the potential for an allergic reaction to cisplatin in a patient allergic to carboplatin. However an allergic reaction to carboplatin is not a contraindication to the use of cisplatin

**Renal Toxicity**

| Renal Toxicity | Nab-Paclitaxel | Carboplatin |
| --- | --- | --- |
| No toxicity during cycle | 100% dose | 100% dose |
| On day of treatment/within 24 hours  Serum Creatinine <10% of  baseline value | 100% dose | Same dose based on baseline creatinine clearance |
| On day of treatment/within 36hours  Serum Creatinine > 10% of baseline value; redetermined creatinine clearance >60 ml/min | 100% dose | Recalculate dose of carboplatin AUC 5 on redetermined GFR and administer if patient otherwise well |
| On day of treatment/within 24 hours  Serum Creatinine > 10% of baseline value; redetermined creatinine clearance 45- 60 ml/min | 100% dose | Recalculate dose of carboplatin AUC 4 on redetermined GFR and administer if patient otherwise well |
| On day of treatment/within 24 hours  Serum Creatinine > 10% of baseline value; redetermined creatinine clearance <45 ml/min | 100% dose | Omit carboplatin |

**Hepatic toxicity**

- If bilirubin level during treatment rises to >1.25 x ULN but < 2 x ULN and transaminases are < 10 x ULN, suggest 25% dose reduction of the nab-paclitaxel.
- Any further rise in bilirubin and /or transaminases despite dose modification above, discontinue nab-paclitaxel.
- Nab-paclitaxel should be re-challenged if bilirubin decreases to <1.25 x ULN and transaminases decrease to < 10 x ULN.
- If the bilirubin level exceeds 3 x ULN and /or transaminases ≥ 10 times ULN then omit that dose of nab-paclitaxel and rechallenge (if applicable) with 25% dose reduction if the bilirubin level falls to <1.25 x ULN and /or transaminases fall to less than 10 x ULN within 7 days.
- If bilirubin <1.25 x ULN and transaminases >ULN but less than 10x ULN continue without dose modification.

**Peripheral neuropathy**

Peripheral neuropathy Grade 2, administer 75% of nab-paclitaxel. For grade 3 or 4 PN, omit nab-paclitaxel.

**Gastrointestinal toxicity**

Patients with Grade 3 or 4 anorexia, nausea, vomiting, constipation, diarrhoea or other gastrointestinal events should be managed as per department protocol.

Hold treatment until symptoms resolve to Grade 1 and then either continue with 100% dose and increase the antiemetic cover or continue with nab-paclitaxel at 80% of the absolute dose and carboplatin at AUC 4 (at the investigator’s discretion).

If there is a further grade 3 occurrence despite dose modifications and maximum antiemetic therapy, discontinue systemic therapy.

**NIVOLUMAB**

| NOTES:  1.                 Severe and life-threatening irAEs should be treated with IV corticosteroids followed by oral steroids. Other immunosuppressive treatment should begin if the irAEs are not controlled by corticosteroids.  2.                 Nivolumab will be permanently discontinued if the irAE does not resolve within 21 days  3.                The corticosteroid taper should begin when the irAE is ≤ Grade 1 and continue at least 2-4 weeks as per treating physician’s discretion.  4.             If Nivolumab has been withheld, it may resume after the irAE decreased to ≤ Grade 1 after corticosteroid taper. | | | | |
| --- | --- | --- | --- | --- |
| irAEs | Toxicity grade (CTCAE v5.0) | Action with Nivolumab | Corticosteroid and/or other therapies | Monitoring and follow-up |
| Pneumonitis | Grade 2 | Withhold^ | ·Administer corticosteroids (initial dose of 1 - 2 mg/kg prednisone or equivalent) followed by taper  Add prophylactic antibiotics for opportunistic infections | ·   Monitor participants for signs and symptoms of pneumonitis  ·   Evaluate participants with suspected pneumonitis with radiographic imaging and initiate corticosteroid treatment |
|  | Recurrent Grade 2, Grade 3 or 4 | Permanently discontinue |  |  |
| Diarrhea / Colitis | Grade 2 or 3 | Withhold^ | Administer corticosteroids (initial dose of 1 - 2 mg/kg prednisone or equivalent) followed by taper | ·   Monitor participants for signs and symptoms of enterocolitis (ie, diarrhea, abdominal pain, blood or mucus in stool with or without fever) and of bowel perforation (ie, peritoneal signs and ileus)  Participants with ≥Grade 2 diarrhea suspecting colitis should consider GI consultation and performing endoscopy to rule out colitis  Participants with diarrhea/colitis should be advised to drink liberal quantities of clear fluids. If sufficient oral fluid intake is not feasible, fluid and electrolytes should be substituted via IV infusion |
|  | Recurrent Grade 3 or Grade 4 | Permanently discontinue |  |  |
| AST or ALT elevation or Increased Bilirubin* | Grade 2 | Withhold^ | Administer corticosteroids (initial dose of 0.5 - 1 mg/kg prednisone or equivalent) followed by taper | ·   Monitor with liver function tests (consider weekly or more frequently until liver enzyme value returned to baseline or is stable) |
|  | Grade 3 or 4 | Permanently discontinue | Administer corticosteroids (initial dose of 1 - 2 mg/kg prednisone or equivalent) followed by taper |  |
| Hypophysitis | Grade 2 | Withhold | Administer corticosteroids and initiate hormonal replacements as clinically indicated | Monitor for signs and symptoms of hypophysitis (including hypopituitarism and adrenal insufficiency) |
|  | Grade 3 or 4 | Withhold or discontinue as per treating physician discretion |  |  |
| Hyperthyroidism | Grade 2 | Continue | Treat with non-selective beta- blockers (eg, propranolol) or thionamides as appropriate | ·   Monitor for signs and symptoms of thyroid disorders |
|  | Grade 3 or 4 | Withhold or  permanently discontinue$ |  |  |
| Hypothyroidism | Grade 2, 3, 4 | Continue | Initiate thyroid replacement hormones (eg, levothyroxine or liothyronine) per standard of care | ·   Monitor for signs and symptoms of thyroid disorders |
| Myocarditis | Grade 1 or 2 | Withhold^ | ·   Based on severity of AE administer corticosteroids | ·   Ensure adequate evaluation to confirm etiology and/or exclude other causes |
|  | Grade 3 or 4 | Permanently discontinue |  |  |
| All Other immune-related AEs | Persistent Grade 2 | Withhold^ | ·   Based on severity of AE administer corticosteroids | ·   Ensure adequate evaluation to confirm etiology or exclude other causes |
|  | Grade 3 | Withhold or discontinue based on the event ^#^ |  |  |
|  | Recurrent Grade 3 or Grade 4 | Permanently discontinue |  |  |
| ^*^AST/ALT: >3.0 - 5.0 x ULN if baseline normal; >3.0 - 5.0 x baseline, if baseline abnormal; bilirubin:>1.5 - 3.0 x ULN if baseline normal; >1.5 - 3.0 x baseline if baseline abnormal;  AST/ALT: >5.0 to 20.0 x ULN, if baseline normal; >5.0 - 20.0 x baseline, if baseline abnormal; bilirubin:>3.0 - 10.0 x ULN if baseline normal; >3.0 - 10.0 x baseline if baseline abnormal; AST/ALT: >20.0 x ULN, if baseline normal; >20.0 x baseline, if baseline abnormal; bilirubin: >10.0 x ULN if baseline normal; >10.0 x baseline if baseline abnormal  ^#^ Events that require discontinuation include but are not limited to: Guillain-Barre Syndrome, encephalitis, Stevens-Johnson Syndrome and toxic epidermal necrolysis.  $ As per the discretion of the principal investigator  ^ Duration of corticosteroids, tapering schedule, and timing of restarting is as per the discretion of the treating physician | | | | |

Any other rare or unexpected adverse events not listed above, but deemed attributable to a study drug, may warrant dose modification or discontinuation at the discretion of the treating physician

1. OUTCOMES

*Primary Outcome*

Surgical conversion rate, defined as the proportion of patients achieving R0 resection following neoadjuvant therapy.

*Secondary outcomes*:

1. *Safety*: Incidence of clinical and laboratory adverse events, graded per CTCAE v5.0. All patients who received at least one cycle of neoadjuvant therapy will be included in safety analyses.
2. *Objective Response Rate (ORR):* Radiological responses will be assessed after 2 cycles according to RECIST v1.1 (based on the sum of diameters (SODs) of the primary tumour (longest diameter) and/or short axis diameter of lymph nodes) by a radiologist and categorized as complete response (CR), partial response (PR), stable disease (SD), or progressive disease (PD).
3. *Pathological Response Rate*: Pathological responses will be evaluated in resected specimens of both primary tumors and lymph nodes. Responses will be classified as pathological complete response (pCR; no residual viable tumor), major pathological response (MPR; ≤10% viable tumor cells), and non-MPR (>10% viable tumor cells).
4. *Survival Endpoints*:

Event-Free Survival (EFS): Time from diagnosis to progression, recurrence, second primary malignancy, or death from any cause. Patients without events will be censored at last follow-up.

Overall Survival (OS): Time from diagnosis to death from any cause. Patients alive at analysis will be censored at last follow-up.

e. *Exploratory biomarkers (Amended Protocol)*

i. *Tumor Immune Microenvironment (TiME) Profiling*:

Pre-treatment profiling will be performed on biopsy specimens. Stromal tumor-infiltrating lymphocytes (sTILs) will be scored as mean percentages across five high-power fields (HPFs). Immunohistochemistry (IHC) quantified CD8⁺ (cytotoxic), FOXP3⁺ (regulatory), and CD4⁺ (helper) T-cell subsets, with immune cell counts to be reported as absolute numbers per HPF Two pathologists will independently assess all slides.

Post-treatment profiling will be performed on resection specimens using the same markers and methodology.

ii. *Volumetric Response Assessment (vRECIST):*

Contrast-enhanced CT scans (2.5 mm slice thickness) and, for oral tongue primaries, fused MRI sequences will be imported into Eclipse v16.1 (Siemens Healthineers, Germany). A head-and-neck–trained radiation oncologist will manually segment the gross tumor volume (GTV) on each scan, delineating the primary lesion and the two largest cervical lymph nodes judged significant by multidisciplinary team (MDT) consensus based on size, morphology, and radiologic features. MDT consensus will be used instead of the strict RECIST short-axis threshold (>1.5 cm) to reflect real-world gross nodal contouring. At each time point, individual GTVs will be summed to derive total tumor volume (TTV). Volumetric response (vRECIST) will be defined as the percentage change in TTV from baseline to after two cycles of neoadjuvant therapy. Response categories will be: vPR, ≥65% decrease in TTV; vSD, change between −65% and <+40%; vPD, ≥40% increase in TTV.

1. **Trial Design and Statistical Analysis**

- **Type of study**: Prospective, single-arm, phase II study.

**Sample Size Determination**

The primary endpoint will be evaluated using Fleming’s two-stage design, testing the null hypothesis of a true surgical conversion rate of 40% against a one-sided alternative of 65%. In stage one, 11 patients will be accrued. Predefined stopping rules for study termination will be applied: the study will stop for futility if ≤5 conversions occur, or the null hypothesis will be rejected early if ≥10 conversions are achieved. Otherwise, 20 additional patients will be accrued to reach a total of 31 evaluable participants. The null hypothesis will be rejected if ≥17 conversions are observed overall, providing a one-sided type I error of 0.0491 and 81% power at a true conversion rate of 65%. A 10% attrition rate will be incorporated, adjusting the target sample size to 34 patients.

**Interim Analysis**

An interim analysis will be performed after completion of stage one (11 patients). The Data Safety Monitoring Board (DSMB) will review the results to determine whether the trial should continue, stop for futility, or reject the null hypothesis early.

**Efficacy Analysis**

The surgical conversion rate (R0 resection rate) will be reported with 95% confidence intervals. Secondary outcomes will include radiological objective response rate (ORR, per RECIST v1.1), pathological response rates (pCR and MPR), and survival endpoints (event-free survival [EFS] and overall survival [OS]). Survival outcomes will be estimated using the Kaplan–Meier method, and group comparisons will be performed using the log-rank test.

**Safety Analysis**

All patients who receive at least one dose of neoadjuvant therapy will be included in the safety population. Adverse events will be summarized as counts and percentages, categorized by CTCAE v5.0 grade and system organ class.

**Exploratory Analyses**

Paired pre- and post-treatment immune biomarker levels will be compared using the Wilcoxon signed-rank test. Correlative analyses between immune markers, volumetric response (vRECIST), and pathological response will be performed using Mann–Whitney U tests, Chi-square tests, or Fisher’s exact tests as appropriate. Correlation coefficients (Spearman’s ρ) will be calculated for associations between continuous variables.

**Statistical Software**

All analyses will be conducted using R.

1. **ETHICAL CONSIDERATIONS**

The study will begin enrolling participants after Institutional Review Board approval and Clinical Trial Registry of India registration. During the study, the investigators will record all adverse events, regardless of treatment or relationship to study drug, as soon as he/she is aware of the event. Investigators will report immediately to the Principal Investigator any serious adverse events. Any serious and or unexpected adverse event will be medically well documented, and the information made available as soon as possible. Serious adverse events will be defined as any event that leads to prolongation of hospital stay, any new disability or death of any of the study participants. This will be reported to the institutional Ethics committee as per standard procedures within the stipulated time from event. All serious adverse events will be reported to the Data Safety Monitoring Board.

Data integrity will be monitored periodically, every 6-12 months. The study team will regularly audit the data by checking data

entered into case report forms with source data to ensure data integrity.
